# Identification of DprE1 inhibitors for tuberculosis through integrated in-silico approaches

**DOI:** 10.1038/s41598-024-61901-x

**Published:** 2024-05-17

**Authors:** Swagatika Dash, Ekta Rathi, Avinash Kumar, Kiran Chawla, Suvarna G. Kini

**Affiliations:** 1 Department of Pharmaceutical Chemistry, Manipal College of Pharmaceutical Sciences, Manipal Academy of Higher Education, Manipal, Karnataka India 576104; 2 Manipal Mc Gill Centre for Infectious Diseases, Prasanna School of Public Health, Manipal Academy of Higher Education, Manipal, Karnataka India 576104; 3 Department of Medical Affairs, Curie Sciences Private Limited, Samastipur, Bihar India 848125; 4 Department of Microbiology, Kasturba Medical College, Manipal Academy of Higher Education, Manipal, Karnataka India 576104

**Keywords:** 3D-QSAR, DprE1, *Mycobacterium tuberculosis*, Molecular dynamics, Pharmacophore, Computational biology and bioinformatics, Drug discovery, Chemistry

## Abstract

Decaprenylphosphoryl-β-D-ribose-2′-epimerase (DprE1), a crucial enzyme in the process of arabinogalactan and lipoarabinomannan biosynthesis, has become the target of choice for anti-TB drug discovery in the recent past. The current study aims to find the potential DprE1 inhibitors through in-silico approaches. Here, we built the pharmacophore and 3D-QSAR model using the reported 40 azaindole derivatives of DprE1 inhibitors. The best pharmacophore hypothesis (ADRRR_1) was employed for the virtual screening of the chEMBL database. To identify prospective hits, molecules with good phase scores (> 2.000) were further evaluated by molecular docking studies for their ability to bind to the DprE1 enzyme (PDB: 4KW5). Based on their binding affinities (< − 9.0 kcal/mole), the best hits were subjected to the calculation of free-binding energies (Prime/MM-GBSA), pharmacokinetic, and druglikeness evaluations. The top 10 hits retrieved from these results were selected to predict their inhibitory activities via the developed 3D-QSAR model with a regression coefficient (R^2^) value of 0.9608 and predictive coefficient (Q^2^) value of 0.7313. The induced fit docking (IFD) studies and in-silico prediction of anti-TB sensitivity for these top 10 hits were also implemented. Molecular dynamics simulations (MDS) were performed for the top 5 hit molecules for 200 ns to check the stability of the hits with DprE1. Based on their conformational stability throughout the 200 ns simulation, hit 2 (chEMBL_SDF:357100) was identified as the best hit against DprE1 with an accepted safety profile. The MD results were also in accordance with the docking score, MM-GBSA value, and 3D-QSAR predicted activity. The hit 2 molecule, (N-(3-((2-(((1r,4r)-4-(dimethylamino)cyclohexyl)amino)-9-isopropyl-9H-purin-6-yl)amino)phenyl)acrylamide) could serve as a lead for the discovery of a novel DprE1 inhibiting anti-TB drug.

## Introduction

Tuberculosis (TB) infection is one of the significant and resurgent public healthcare issues worldwide, with a high incidence and mortality rate. In recent years, it has been aggravated due to its association with undernutrition, poverty, and its comorbidity with HIV/AIDS, which permits the reactivation of latent TB infection^1^. As per the World Health Organization (WHO) latest Global Tuberculosis Report (2022), TB affected approximately 10.6 million people and killed 1.6 million lives in 2021^2^. The dramatic upsurge in the prevalence of drug-resistant tuberculosis (DR-TB) during the last three decades also exacerbates the TB burden globally. Multidrug-resistant TB (MDR-TB), expressed as the resistance to two front-line drugs, rifampicin and isoniazid, needs a duration of 6–9 months of treatment. This treatment period can extend up to 12 months or beyond in case of pre-extensively drug-resistant TB (pre-XDR-TB), defined as MDR-TB with additional resistance to fluoroquinolone (FQ) and XDR-TB, described as MDR-TB plus resistance to any FQ and moxifloxacin, levofloxacin, bedaquiline, and linezolid^3^. This consistent threat posed by tuberculosis has demanded a revival of interest in developing new anti-TB antibiotics. An intensive understanding of the druggable targets in the Mtb bacillus is a prerequisite to underpin this progress. Among the several molecular targets in Mtb, Decaprenylphosphoryl-β-d-ribose-2′-epimerase (DprE1) has become one of the emerging targets in anti-TB drug discovery research in the recent past^4^. From the outcome of prior research, diversified chemical scaffold-bearing compound libraries were identified as DprE1 inhibitors, and four of these molecules (BTZ-043, Macozinone/PBTZ-169, TBA-7371 & OPC-167832) are in the clinical development stages^5,6^. These advancements authenticate the druggability of the DprE1 target and its significance in TB drug development, which inspired us to conduct this study. Now, the question comes to one’s mind as to why it has become the target of interest for researchers and what its role is in the cellular mechanism of Mtb. To address this question, we briefly outline its detailed mechanism of action here.

**Figure 1:**
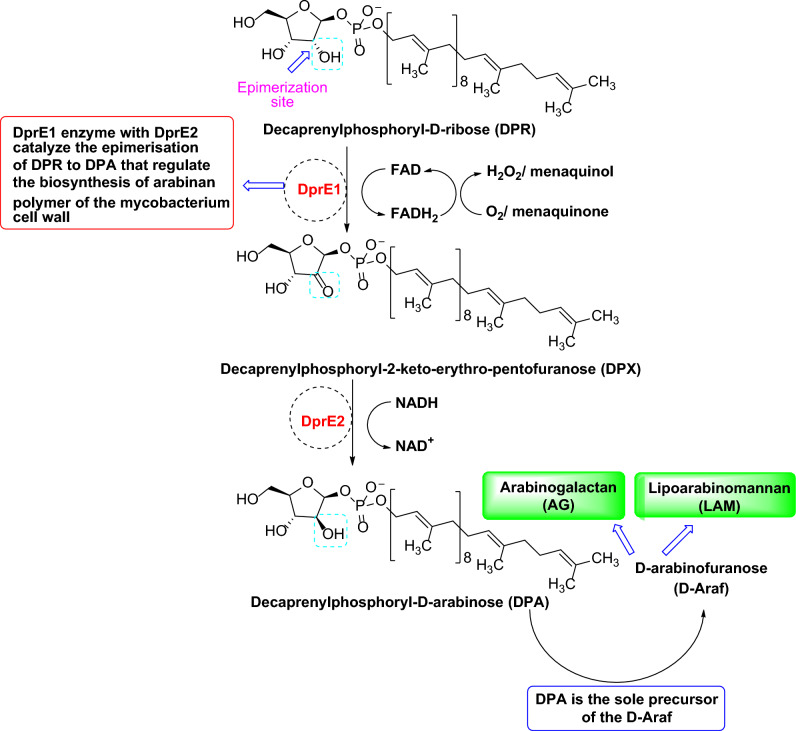
DprE1/DprE2 catalyzed epimerization reaction of ribose to form arabinose at the site of the 2′-OH group of DPR.

Decaprenylphosphoryl-β-d-ribose-2′-epimerase (DprE1) is one of the enzyme machinery that catalyzes the biosynthetic pathway for the production of the two crucial heteropolysaccharide structure of Mtb cell envelope, arabinogalactan (AG) and lipoarabinomannan (LAM). LAM and AG consist of a typical sugar moiety, d-arabinofuranose (d-Araf). And decaprenylphosphoryl-d-arabinose (DPA) is the sole donor of d-Araf. The metabolic pathway involved in the synthesis of DPA comprises the conversion of the sugar moiety, ribose, to arabinose. This includes two crucial steps of epimerization at the C-2′ hydroxyl group of ribose moiety^7^. DprE1 enzyme catalyzes the oxidation of decaprenylphosphoryl-d-ribose (DPR) to form the intermediate, decaprenylphosphoryl-2′-keto-ribose (DPX) with the reduction of FAD cofactor to FADH_2_. Subsequently, the intermediate DPX is reduced to DPA by using NADH-dependent deceprenylphosphoryl-2-keto-ribose reductase (DprE2). The reduced form of the FAD cofactor, FADH_2,_ has to be oxidized through an electron acceptor like menaquinone or molecular oxygen to assist the new epimerization cycle, depicted in Fig. 1^8^. Also, this epimerization step takes place in the periplasmic localization of the Mtb cell wall, elucidating the vulnerable character of DprE1 by developing an enormous number of inhibitors with diverse heterocyclic ring scaffolds^9^. According to their mechanism of inhibition, these inhibitors are classified as (a) covalent inhibitors, which inhibit the DprE1 enzyme irreversibly by making a covalent adduct with Cys387 amino acid, and (b) non-covalent inhibitors, which work as competitive inhibitors. More than 15 chemical scaffolds of DprE1 (both covalent and non-covalent) inhibitors have been discovered^10^. Despite these, issues like toxicity related to covalent inhibitors and less in-vivo efficacy of non-covalent inhibitors need new arsenals targeting the DprE1 enzyme to fight against Mtb in the war against TB.

Drug discovery is the initial stage of the drug development procedure, and the application of computational methods in drug discovery fast tracks and economizes this process through various tools. Virtual screening is a type of notion in drug discovery that uses computational strategy to determine the potential target binders. Two screening methods are employed in drug discovery, i.e., Ligand-based (LB) screening and Structure-based (SB) screening^11^. In the SB approach, the compounds from databases were obtained to dock with a protein target and to find the best-fitted compound. However, based on knowledge about the known active molecules, the second type of approach is the LB approach. In this approach, the active chemicals were utilized to generate Quantitative Structure–Activity Relationship (QSAR) and pharmacophore models, which identify the required structural features of a molecule for binding to the specified target^12^. In this study, we used both LB-pharmacophore and SB-molecular docking screening methods to find the best hits. LB-Pharmacophore models and 3D-QSAR model were generated using the reported azaindole-based DprE1 inhibitors. The top pharmacophore model was applied to screen the molecules. The resulting screened compounds were considered for rigid docking and free binding energy (Prime/MM-GBSA) methods, followed by ADME/T evaluation. Again, the interpretation of anti-TB sensitivity and DprE1 inhibition of the top selected hits was predicted. Finally, the induced fit docking (IFD) and molecular dynamics (MD) approaches were carried out to establish the binding potential of the top hit molecules. The overall In-silico procedures applied for this analysis might help in identifying potent inhibitors against DprE1 for TB.

## Materials and methods

**Figure 2:**
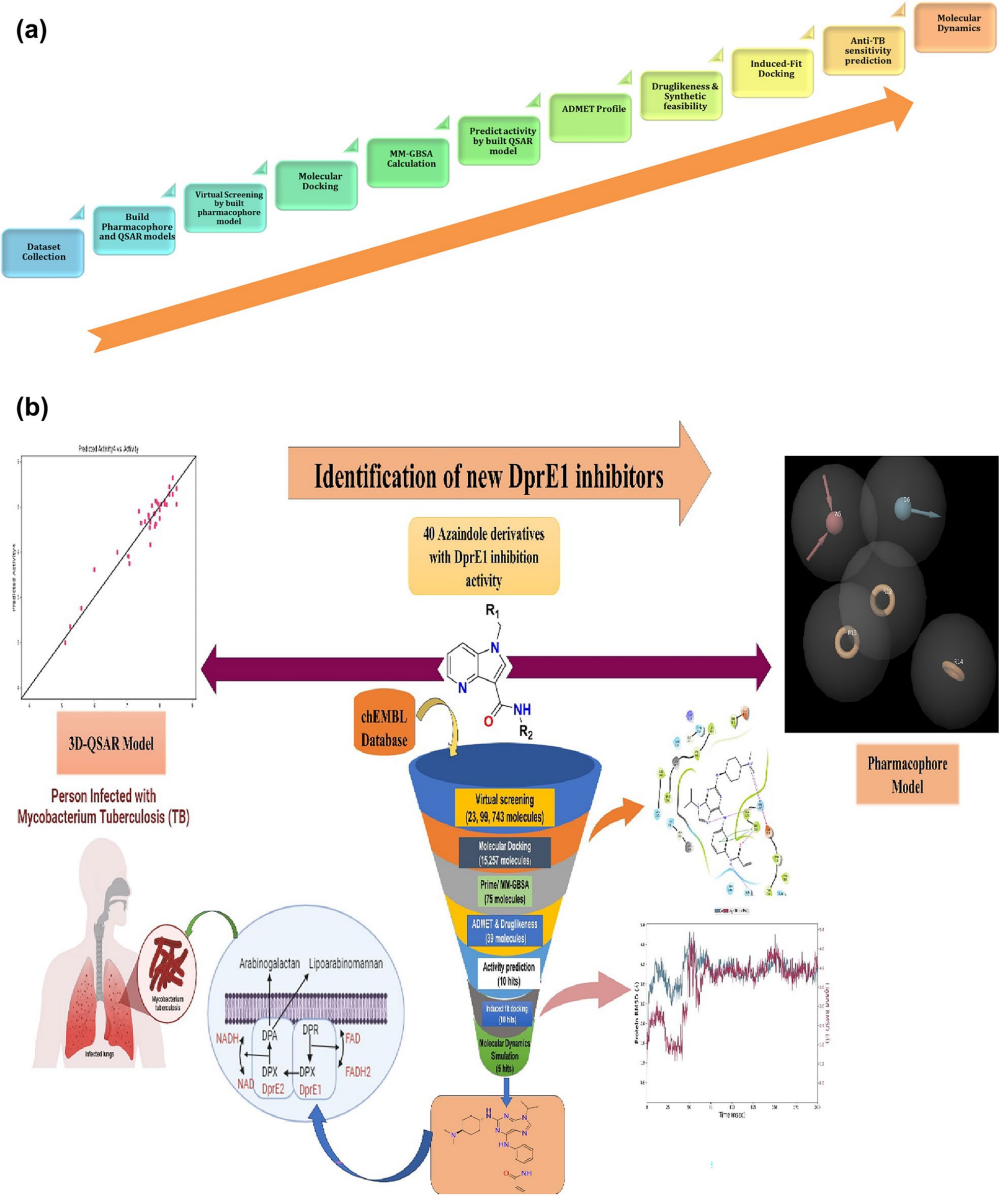
(**a**) Workflow of the study, (**b**) Flowchart of *in-silico* methods employed in the current study.

All the molecular modelling studies were accomplished by utilizing the commercial Schrodinger Drug Discovery Suite (Maestro Version 11.8) installed on an HP desktop system with the Linus Ubuntu operating system. This comprises “QSAR and Pharmacophore development” using the Phase module, “Ligand docking” using the Glide tool, “Induced-Fit Docking” and “MM-GBSA” (binding energy calculation) using the Prime module, and “Molecular dynamics” using the Desmond tool. The ADME profiles were assessed by utilizing the Maestro QikProp module. Further, freely available online software like pkCSM, SwissADME, and mycoCSM was used to predict toxicity, synthetic accessibility, and anti-mycobacterial properties of the selected hits. Figure 2a indicates the workflow, and Fig. 2b briefly depicts the in-silico methodology adopted in the current study.

### Preparation of datasets

An assemblage of 40 azaindole derivatives of DprE1 inhibitors collected from the literature was considered^13,14^, depicted in Supplementary Table S1. Before selecting the dataset, the indispensable requirement was that the biological activity should be determined using the same assay protocol for all the compounds. The above specification was fulfilled by the selected dataset to execute the studies. The structures for the compounds were drawn by employing the 2D sketcher drawing tool existing in Maestro (Builder panel) and converted into the form of 3D. Their energy minimizations were carried out using the LigPrep panel of Maestro by adding partial atomic charges and retaining the specific chirality with the generation of ionization forms. All these processes were conducted by employing the OPLS3e force field. The biological activities (DprE1 enzyme inhibition) of these compounds expressed in the form of IC_50_ in the micromolar range were converted to pIC50 by applying the formula pIC50 = log_10_ [IC_50_ (µM) × 10^–6^]. The pIC50 values of all the compounds were considered for deriving both pharmacophore and QSAR models.

### Generation and validation of the pharmacophore model

A pharmacophore is a framework of the steric and electronic characteristics of the molecules that are essential to establish the optimum molecular interactions with the particular biological target for showing biological responses^15^. The generation of pharmacophore hypotheses, which can be constructed using either the structure of the biological target or its inhibitors, makes it relevant to identify new scaffolds. This method is based on the common steric and electronic characteristics of 3D structures required to exhibit biological response^16^. Maestro (Schrodinger) software containing the PHASE module constructed the pharmacophore hypotheses using multiple ligands of the collected dataset^13,14^. The PHASE module with the ‘Develop Pharmacophore Hypothesis’ option was utilized to build the pharmacophore model by applying the tree-based separation technique to determine pharmacophoric features^17^. The dataset of compounds selected was assigned as active and inactive on the basis of their biological activity (pIC50). In this dataset of compounds, the ligands with a pIC50 value above 8 and the ligands with a pIC50 value below 7 were determined as active and inactive molecules, respectively, and the remaining ligands having pIC_50_ values between 7–8 were taken as moderately active (Supplementary Table S1). Six features of hypotheses, i.e., H-bond acceptor (HBA), H-bond donor (HBD), hydrophobic groups (H), aromatic ring (R), negative and positive ionizable groups (N/P) were computed for building the pharmacophore models. The site points with a minimum of four and maximum of five and the inter-site distance of a maximum of 2.0 Å were fixed during the generation of the common pharmacophore hypotheses with the highest optimal probable combinations. Among the built pharmacophore models, the best models were selected according to their survival score, site score, volume score, vector score, and BEDROC score^18^. The selected pharmacophore hypotheses were further validated through enrichment calculation.

Validation is required to obtain accurate pharmacophore analysis and assess the generated model's quality. Hence, these built pharmacophore models were validated to check the reliability of these models. To perform the validation of the models, the retrieved Schrodinger decoy dataset and the active compounds of this study were subjected to determine the model's predictive capability in recognizing active and inactive molecules. Further, the models were ranked and validated by their Phase Hypo score, BEDROC160.9, early enrichment factor (EF1%), AUAC (Area Under the Accumulation Curve), ROC (Receiver Operator Characteristics area under the curve), and Average Outranking Decoys values computed through enrichment calculation^19^.

### Atom-based 3D QSAR model generation

The 3D-QSAR model depicts the necessary structural features needed to regulate the biological actions of the compounds and the regions responsible for increased and decreased biological activity. This statistical information is being used to predict the activities of new chemical compounds and to design new leads with improved activity^20^. The same database of DprE1 inhibitors used for pharmacophore modeling was utilized to develop the 3D QSAR model. The developed QSAR model was employed for the prediction of activities of the newly screened molecules. The PHASE module carrying the atom-based QSAR model option in the Maestro panel was employed to build the 3D atom-based QSAR model for the prediction of the DprE1 inhibitory activity of the screened compounds. Initially, the dataset of inhibitors was randomly split into the training and test sets in a 70:30 ratio. Grid spacing was fixed to 1 Å, and the Partial Least Square (PLS) factor was put to 4 for the generation of the QSAR model^21^. The quality and stability of the model were determined by individually calculated components such as standard deviation (SD), regression coefficients (R^2^), cross-validated R^2^ (R^2^ CV), stability (S), F-value, P-value, root mean square error (RMSE), predictive correlation coefficient (Q^2^), and Pearson-r. The leave-one-out (LOO) strategy was utilized for internal validation using the training set that computes R^2^ and R^2^ CV. Similarly, the test set was employed to validate the model's predictive power externally by computing Q^2^ and other components^22^.

### Virtual screening by generated pharmacophore model

From the constructed pharmacophore hypotheses, the best hypothesis was selected and employed as a 3D search query for the virtual screening of the chEMBL database (https://www.ebi.ac.uk/chembldb) of 23,99,743 molecules. It is a manually curated repository of diversified bioactive molecules extracted from several peer-reviewed journals possessing druglike characteristics. This database is easily accessible and high-quality because of its manual curation and includes bioactivity data from each stage of the drug development process^23^. Prior to the screening, the collected compounds from databases were optimized into their lowest energy 3D conformations by the LigPrep module. The panel of Phase ligand screening was used to retrieve compounds as DprE1 inhibitors from the databases^24^. These screened compounds were selected by their phase fitness score and the number of matches to the chemical features.

### Structure-based screening of pharmacophore-based screened hits (molecular docking)

After the screening through pharmacophore models, we preferred the compounds with top fitness scores for further filtration through molecular docking into the binding spaces of the DprE1 structure. The crystal structure of the DprE1 enzyme (PDB ID: 4KW5) was recovered from the RCSB-PDB databank (https://www.rcsb.org/structure/4KW5). This crystal structure was put for protein preparation by utilizing Maestro's Protein Preparation Wizard module. In this process, missing H-atoms were added, and the ionization state of the metal was fixed to maintain structural correctness. The protein preparation wizard module has three tools: preprocessing, reviewing and modifying, and refining to process and minimize the 4KW5.pdb structure. In the workflow of protein preparation, the protein PDB structure was first imported and processed by assigning bond orders, adding hydrogens, filling missed side chains by prime, and optimizing pH. This preprocessed protein was further modified by deleting some extra chain and hetero atoms for the following refinement process. The refinement step optimized the H-bonds of protein by reducing its preparation time and minimized the protein that allows the free movement of ligands inside it^25^.

The receptor grid is a type of search space that represents the shape and properties of the receptor by various force fields and provides more reliable scoring results. The grid generation method is mainly based on the ligand binding sites, which identify the active sites in the protein structure. The centroid of the workspace ligand provides a precise representation of the active site’s location. This ensures that the generated receptor grid accurately encompasses the binding pocket where ligands are expected to interact with the target protein. Also, for the validation of the docking protocol, the bound ligand of the receptor had to be redocked to its original binding site. Therefore, the receptor grid was generated using the binding site of the co-crystallized ligand of the DprE1 protein.

Here, a cube-shaped grid was generated by utilizing the Receptor Grid generation panel of the GLIDE module by picking the centroid of the workspace ligand present at the active space of DprE1 protein (4KW5). The van der Waals radii of the receptor atoms were scaled up with a partial charge cutoff value of 0.25 and a scaling factor of 1.0^26^. Eventually, the ligand-receptor docking was performed for all the preferred hits by using the generated grid through the ligand docking workflow of the Glide module. This process investigates the inhibition potential of the studied candidates against the target enzymes and finds the optimum conformation of the formed complex between the selected ligands and a receptor. The different docking modes, like HTVS (High-Throughput Virtual screening) followed by SP (Standard Precision) and XP (extra precision) modes, were applied here for performing computational docking^27^. The docking protocol was validated by first removing the inhibitor from the complex, then re-docking it, and finally calculating the root mean square deviation (RMSD). The resulting docking scores and binding interactions of the compounds were compared with the docked score of co-ligand, along with its crucial amino acid residue interactions. The compounds with higher docking scores were filtered again for the following process.

### MM-GBSA calculation of the docked complexes

The approach of molecular mechanics, along with generalized Born and surface area solvation (MM-GBSA), is applied to estimate the absolute ligand–protein binding affinities by calculating its free binding energy. The prime module containing the MM-GBSA panel with default settings was used for estimating the free-binding energy. The docked molecules-receptor complex with a top Glide XP docking score was considered to perform this MM/GBSA task^28^. The formula employed for computing the binding free energies of the ligands is mentioned below.

$$\Delta {\text{G}}_{{({\text{binding}})}} = \, \Delta {\text{E}}_{{({\text{MM}})}} + \, \Delta {\text{G}}_{{({\text{solvation}})}} + \, \Delta {\text{G}}_{{({\text{SA}})}}$$

$${\text{MM}}/{\text{GBSA}}_{{({\text{dG}}\;{\text{bind}})}} = {\text{ G}}_{{({\text{Optimized}}\;{\text{Complex}})}} - {\text{ G}}_{{({\text{Optimized}}\;{\text{Free}}\;{\text{Receptor}})}} - {\text{ G}}_{{({\text{Optimized}}\;{\text{Free}}\;{\text{Ligand}})}}$$

where ΔG_(binding)_ is mainly the difference between the polar desolvation energies, ΔE_(MM)_ is basically the difference between the minimized energies of the DprE1-hit molecule docked complex, and the sum of the individually calculated minimized energies of the DprE1 structure and the corresponding hit. Similarly, ΔG_(solvation)_ is the difference in the GBSA solvation energy of the ligand-DprE1 complex and the summation of the solvation energies of the DprE1 enzyme structure and ligand. Whereas ΔG_(SA)_ is a change in the surface area energies of the bounded complex and the total surface area energies of the unaligned receptor and picked hits. The solvation model of VSGB combined with the variable-dielectric generalized Born model under the OPLS3e force field calculated the ligand binding energy in the Prime MM-GBSA method^29^.

### Pharmacokinetic (PK) and toxicity analysis

Together with the optimum binding affinities of the molecules with the target, the potency of the hit molecules is another driving factor in the drug development process. To become therapeutically successful and effective like drugs, the hits must possess high biological actions with low toxicity. The evaluation of ADMET (A: Absorption, D: Distribution, M: Metabolism, E: Excretion, T: Toxicity) properties of small molecules experimentally is high-priced and time-consuming. Therefore, the approach of computational evaluation of pharmacokinetic (PK) and toxicity profiles of small molecules has been fostered as an alternative in the initial stage of drug development. The ADME profiles of the hit molecules were assessed by using the Maestro QikProp module. The job was run in default setting mode with the use of VSGB 2.0 as a solvation model in OPLS_ 2005 forcefield^30^. Various parameters such as molecular weight (MW), the no. of hydrogen bond donors (HBD), the no. of hydrogen bond acceptors (HBA), octanol–water partition coefficient, solubility, HERG K^+^ channel blockage prediction, the permeability of Caco-2 cell line, Blood–Brain-Barrier (BBB) partition coefficient, CNS active prediction, and human oral absorption were determined. These parameters help consider a small molecule as a drug candidate^31^.

The lead candidates were also evaluated for in-silico toxicity prediction through pkCSM (https://biosig.lab.uq.edu.au/pkcsm/), an online tool with the aim of effective and safe drug delivery. This pkCSM approach is based on distance-related graph signatures that predict the PK and toxicity properties of the molecules by constructing 30 predictors. The compounds in the SMILE format were used as input files for calculating various ADMET parameters^32^. In this study, both organ and genome toxicities were checked by calculating various toxicity parameters such as AMES toxicity, the maximum amount of tolerated dose for humans, oral rat acute toxicity in the form of LD_50,_ and oral rat chronic toxicity in the form of LOAEL (Lowest Observed Adverse Effect)^33^.

### Druglike properties and synthetic feasibility prediction

The lead compounds were further subjected to estimate their druglike properties by using five diverse rule-based filters such as Lipinski filter (Pfizer), Ghose filter (Amgen), Verber filter (GSK), Egan filter (Pharmacia), and Muegge filter (Bayer). Additionally, bioavailability was also determined by employing the Abbott bioavailability scale. Later, the selected hits were put in for the prediction of structural alerts like PAINS and Brenk alerts. Further, leadlikeness and synthetic accessibility were also derived for those molecules^34^. All these predictions were accomplished via the web server, SwissADME (http://www.swissadme.ch/), by submitting the compound’s structure in SMILE format^35^.

### Induced fit docking (IFD)

Simultaneously, after ligand binding, the proteins generally experience movements either inside the chain or in the backbone. However, this is not possible in conventional docking procedures, which may show misleading results. The flexible docking or IFD methodology was utilized to overcome the problem. This IFD is a mixed approach of molecular docking and molecular dynamics, commonly applied to cross-validate the affinity of the selected molecules with the target receptor^36^. Here, the IFD process was conducted by utilizing the Prime IFD panel of the Maestro. In IFD, the best molecules were rigidly docked in the flexible receptor of DprE1 by XP GLIDE mode. During the process of IFD, the Van der Waals scaling value of 0.50 and the maximum 20 ligand poses were adjusted for every ligand. Later, the generated poses were subjected to the estimation of the sidechain and for minimization through the refinement of the backbone within a 5.0 Å distance. Finally, the IFD scores were calculated for the individual pose, and each ligand's highest negative IFD score was considered for further molecular dynamics study^37^.

### Anti-tubercular sensitivity prediction

The traditional process of testing the drugs against Mtb is challenging due to the slower evolution of bacilli. Nowadays, machine learning (ML) based insilico tools help predict the anti-tubercular sensitivity of compounds on a prior basis, which would substantially reduce drug development cost and time. In this study, we used the online server mycoCSM (https://biosig.lab.uq.edu.au/myco_csm/prediction) for the prediction of the anti-TB activity of the selected hits^38^. The SMILES format of the compounds was uploaded as input form, and the analysis report was downloaded in a .csv format^39^.

### Molecular dynamics simulation (MDS) investigation

The ligand-DprE1 docked complex with the highest IFD score was further selected for molecular dynamics (MD) simulation analysis to determine the stability of the complex over time. MD is more relevant than molecular docking as it allows flexible interaction between receptor and ligand through the solvation of the system. This analysis was performed utilizing the Desmond MD tool in the Maestro software. A three-step process of system builder, minimization, and MD simulation completed the MD job. During the system-building step, the ligand-receptor docked complex was solvated utilizing a simple point charge (SPC) water model and contoured inside an orthorhombic-shaped box having dimensions 10 × 10 × 10 Å. To neutralize the system, 0.15 M concentrations of counter-ions such as Na^+^ and Cl^−^ were added, mimicking the body’s physiology. The next step of minimization was done for the solvated complex with 2000 maximum iterations and 1 kcal/mol/Å convergence threshold^40^. This minimization process mainly relaxes the complex into minimum energy. The last step was MD simulation, which primarily simulated the Newtonian dynamics of the complex system under the selected ensemble conditions of NPT, i.e., normal pressure and temperature. The OPLS_2005 force field was employed to carry out the simulation task by setting the temperature at 300 K and pressure at 1.01325 bar. Nose–Hoover and Martina–Tobias–Klein methods were used as default settings for thermostat and barostat, respectively, to control the temperature and pressure^22^. The simulation was performed for 200 ns, and the recording time gap was set to 200 ps. At an interval of every 200 ps, all the new coordinates, velocities, and energies were saved. The simulation interaction diagram panel was utilized to generate the output MD data. The output file containing RMSD and RMSF plots, ligand interaction, and histogram were used to analyze the simulation results and observe the docked complex's stability.

## Results

### Ligand-based pharmacophore hypothesis development and 3D QSAR modeling studies

#### Validation of established pharmacophore hypotheses

**Figure 3:**
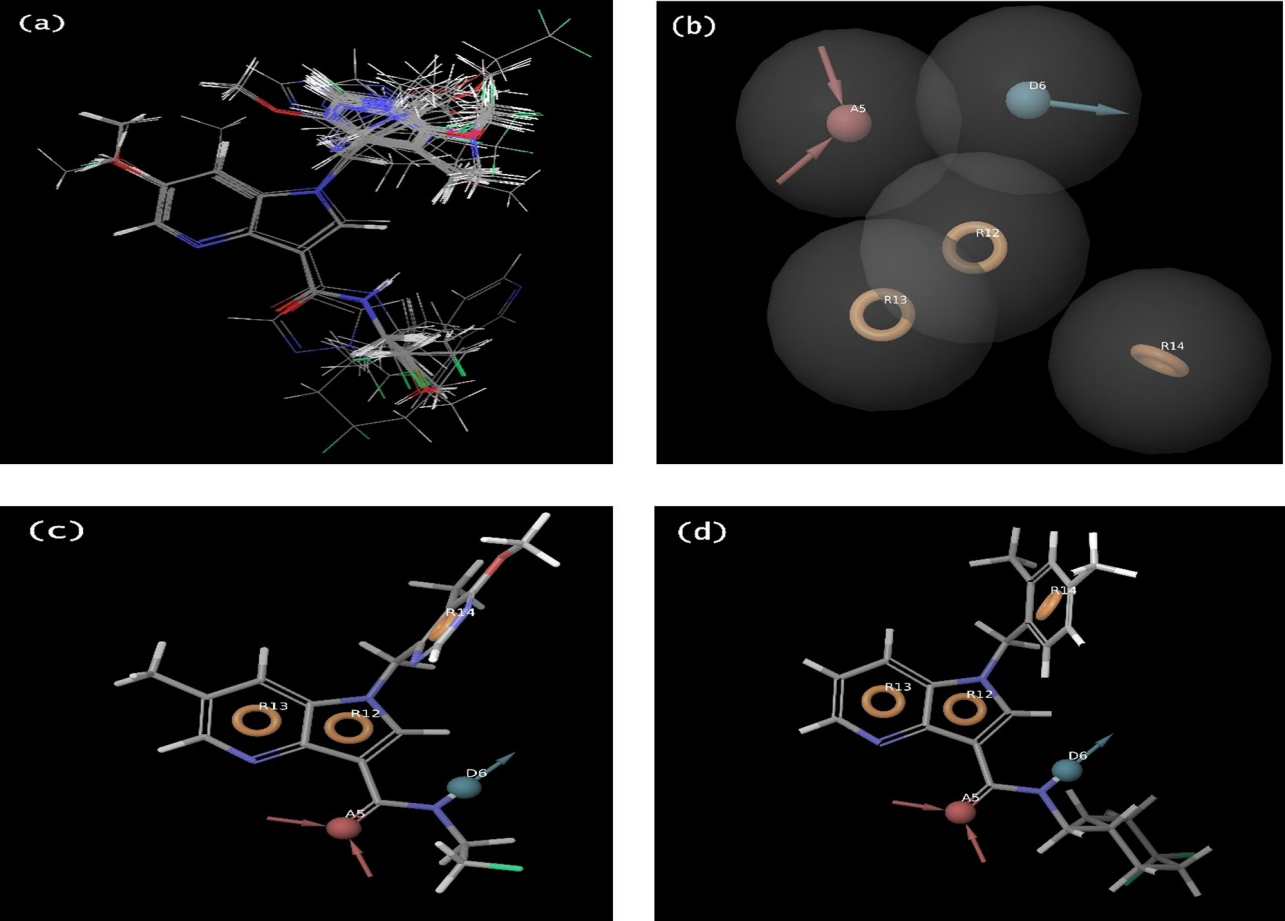
(**a**) Structural alignment of collected 40 DprE1 inhibitors chosen for both pharmacophore and QSAR modeling. (**b**) The selected pharmacophore hypothesis ADRRR_1 with its pharmacophoric features, one H-bond acceptor (A5), one H-bond donor (D6), and three aromatic rings (R12, R13, R14), displayed individually with pink colour sphere, cyan colour sphere, and orange colour torus cubic sites. (**c**) The alignment of ADRRR_1 hypothesis on the most active compound 4. (**d**) The alignment of ADRRR_1 hypothesis on the most active compound 11.

In this present investigation, a series of 40 azaindole moiety-containing DprE1 inhibitors chosen for both QSAR and pharmacophore modeling studies were imported together with their reported activities (Supplementary Table S1)^13,14^. Next, the prepared low conformers of 40 DprE1 inhibitors were submitted to ligand alignment, which was further employed to build both models. For the alignment of molecules, the flexible ligand alignment (FLA) module, which has a standard scaffold alignment option, was employed. This FLA tool used minimized conformers for perfect alignment by employing the popular Bemis-Murcko method, displayed in Fig. 3a ^26^.

A total of 20 different combinations of 10 five-point and 10 four-point pharmacophore hypotheses were generated by the PHASE module of Maestro. The enrichment report of all the generated pharmacophore hypotheses was provided in the Supplementary Table S2. Among all the hypotheses, two five-point hypotheses (ADRRR_1) and (ADRRR_3) showed better survival scores than others (Table 1). But the ADRRR_1 (Fig. 3b) hypothesis clearly defined actives over inactives in the used DprE1 dataset than ADRRR_3, which was identified through their alignment with the most active and least active compounds (Supplementary Fig. 1). The alignment of hypothesis ADRRR_1 with the most active (compound 4) and the least active (compound 11) compounds (Supplementary Table S1), as depicted in Fig. 3c and d.

**Table 1:** Developed pharmacophore models in terms of their scoring parameters.

Sl. No.	Hypothesis	Survival score	Site score	Vector score	Volume score	BEDROC score	Phase hypo score
1	AAADR_1	4.868	0.839	0.951	0.741	1.000	1.292
2	**ADRRR_1**	**5.484**	**0.995**	**0.984**	**0.856**	**0.998**	**1.327**
3	ADRRR_2	5.477	0.992	0.987	0.849	0.993	1.322
4	ADRRR_3	5.420	0.972	0.992	0.811	1.000	1.325
5	ADRRR_4	3.683	− 0.098870	0.676	0.459	0.970	1.191
6	AADRR_1	5.294	0.995	0.984	0.856	0.998	1.316
7	AADRR_2	5.287	0.992	0.987	0.849	0.995	1.312
8	AADRR_3	5.224	0.971	0.993	0.810	1.000	1.313
9	AADRR_4	5.062	0.827	0.948	0.750	1.000	1.303
10	AADRR_5	4.979	0.832	0.938	0.729	0.983	1.282
11	ADRR_1	5.068	0.994	0.980	0.856	0.995	1.299
12	ADRR_2	5.066	0.994	0.980	0.856	0.998	1.302
13	ADRR_3	5.060	0.992	0.984	0.849	0.993	1.297
14	ADRR_4	5.044	0.995	0.980	0.855	0.996	1.299
15	ADRR_5	5.042	0.992	0.984	0.831	0.987	1.289
16	ADRR_6	5.032	0.999	1.000	0.857	1.000	1.301
17	ARRR_1	5.096	0.994	0.980	0.856	0.995	1.301
18	ARRR_2	5.090	0.992	0.984	0.849	0.985	1.290
19	DRRR_1	5.202	0.994	0.980	0.856	0.995	1.307
20	DRRR_2	5.149	0.982	0.989	0.809	1.000	1.308

Significant values are in bold.

*Site score* to measure the alignment of the site points, calculated by align score (a_s_) and align cutoff (a_c_). Site score = 1 − a_s_/a_c_. Ranges from o to 1.

*Vector score* average cosine angles formed by vector features (A, D, R) pairs. Value is between − 1 to 1.

*Volume score* to measure the volume contributed by the structures during alignment on the pharmacophore. The range limit is between 0 to 1.

*BEDROC score* to measure the differentiation power of the hypothesis for selecting active compounds from decoys. Value is from 0 to 1 (ideal).

*Phase hypo score* ranking the hypotheses for their performance during virtual screening and also for perfect ligand aligning.

*Survival score* to appraise the alignment quality and calculated by employing all the above values. S = 1 × site score + 1 × vector score + 1 × volume score + 1 × selectivity score + 1 × log_10_ (No. of matches) − 1 × inactive score.

**Figure 4:**
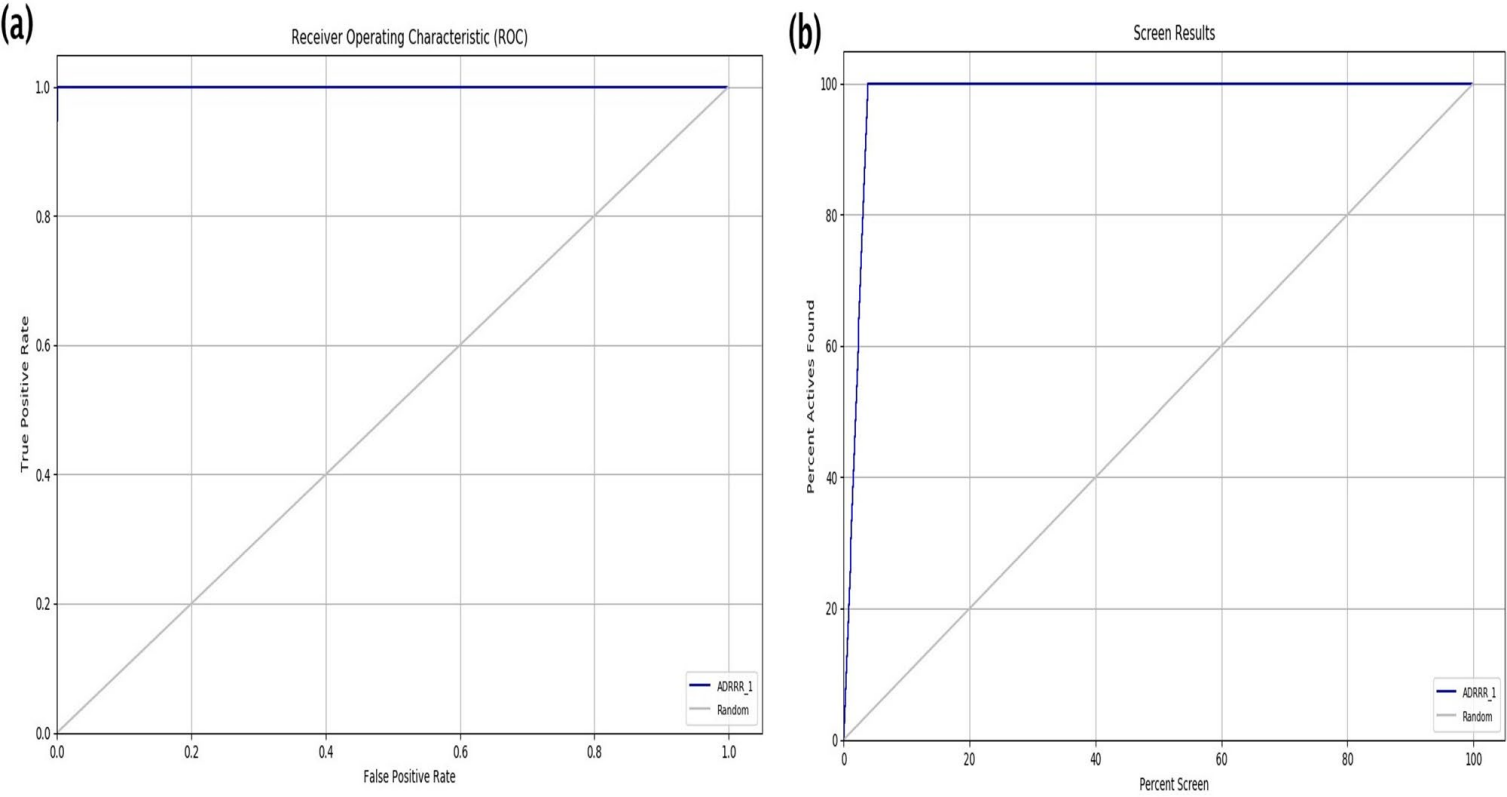
(**a**) Receiver Operating Characteristic (ROC) plot. (**b**) Screening plot of ADRRR_1 hypothesis.

The top hypothesis, ADRRR_1, was further confirmed for screening the database on the basis of its enrichment report calculated using active and decoy sets retrieved from Schrödinger (Table 2)^19^. The selected model with pharmacophoric features of ADRRR_1 showed the highest Phase Hypo score of 1.33 among all the models, which indicated the model's better differentiation ability among active and inactive ligands. The enrichment factor or EF% was 26.64, with an excellent BEDROC score of 1. The enrichment calculation also confirmed the quality and accuracy of the pharmacophore models with an excellent ROC value of 1, AUAC value (0.98), and average outranking decoys of 0.00. Generally, the Receiver Operator Characteristics area under the curve (ROC) gives information about the degree of separation, whereas AUAC determines the model's performance. The ROC plot and screening plot of the ADRRR_1 hypothesis, provided in Fig. 4a and b, indicate the reliability and repeatability of the model.

**Table 2:** Enrichment report to rank the generated pharmacophore hypotheses during validation.

Serial no.	Hypothesis	Phase hypo Score	EF%	BEDROC 160.9	ROC	AUAC	Avg outranking decoys	Matches
1	AAADR_1	1.29	26.64	1.00	1.00	0.98	2.59	4 of 5
2	AADRR_1	1.32	26.64	1.00	1.00	0.98	0.05	4 of 5
3	AADRR_2	1.31	5.33	0.27	0.81	0.80	187.51	4 of 5
4	AADRR_3	1.31	26.64	0.98	0.99	0.97	11.62	4 of 5
5	AADRR_4	1.30	26.64	1.00	1.00	0.98	3.41	4 of 5
6	AADRR_5	1.28	21.31	0.82	0.95	0.93	49.92	4 of 5
7	**ADRRR_1**	**1.33**	**26.64**	**1.00**	**1.00**	**0.98**	**0.00**	**4 of 5**
8	ADRRR_2	1.32	2.66	0.17	0.84	0.82	163.00	4 of 5
9	ADRRR_3	1.33	15.98	0.68	0.98	0.97	15.46	4 of 5
10	ADRRR_4	1.19	0.00	0.00	0.71	0.70	290.72	4 of 5
11	ADRR_1	1.30	26.64	1.00	0.95	0.95	0.00	4 of 4
12	ADRR_2	1.30	23.98	0.95	0.95	0.95	0.81	4 of 4
13	ADRR_3	1.30	2.66	0.09	0.90	0.90	51.24	4 of 4
14	ADRR_4	1.30	26.64	1.00	0.95	0.95	0.00	4 of 4
15	ADRR_5	1.29	26.64	0.94	0.91	0.91	43.76	4 of 4
16	ADRR_6	1.30	26.64	1.00	0.97	0.97	0.00	4 of 4
17	ARRR_1	1.30	26.64	1.00	0.97	0.97	0.00	4 of 4
18	ARRR_2	1.29	2.66	0.15	0.95	0.94	27.08	4 of 4
19	DRRR_1	1.31	26.64	1.00	0.95	0.95	0.11	4 of 4
20	DRRR_2	1.31	2.66	0.18	0.50	0.71	19.20	4 of 4

Significant values are in bold.

**Figure 5:**
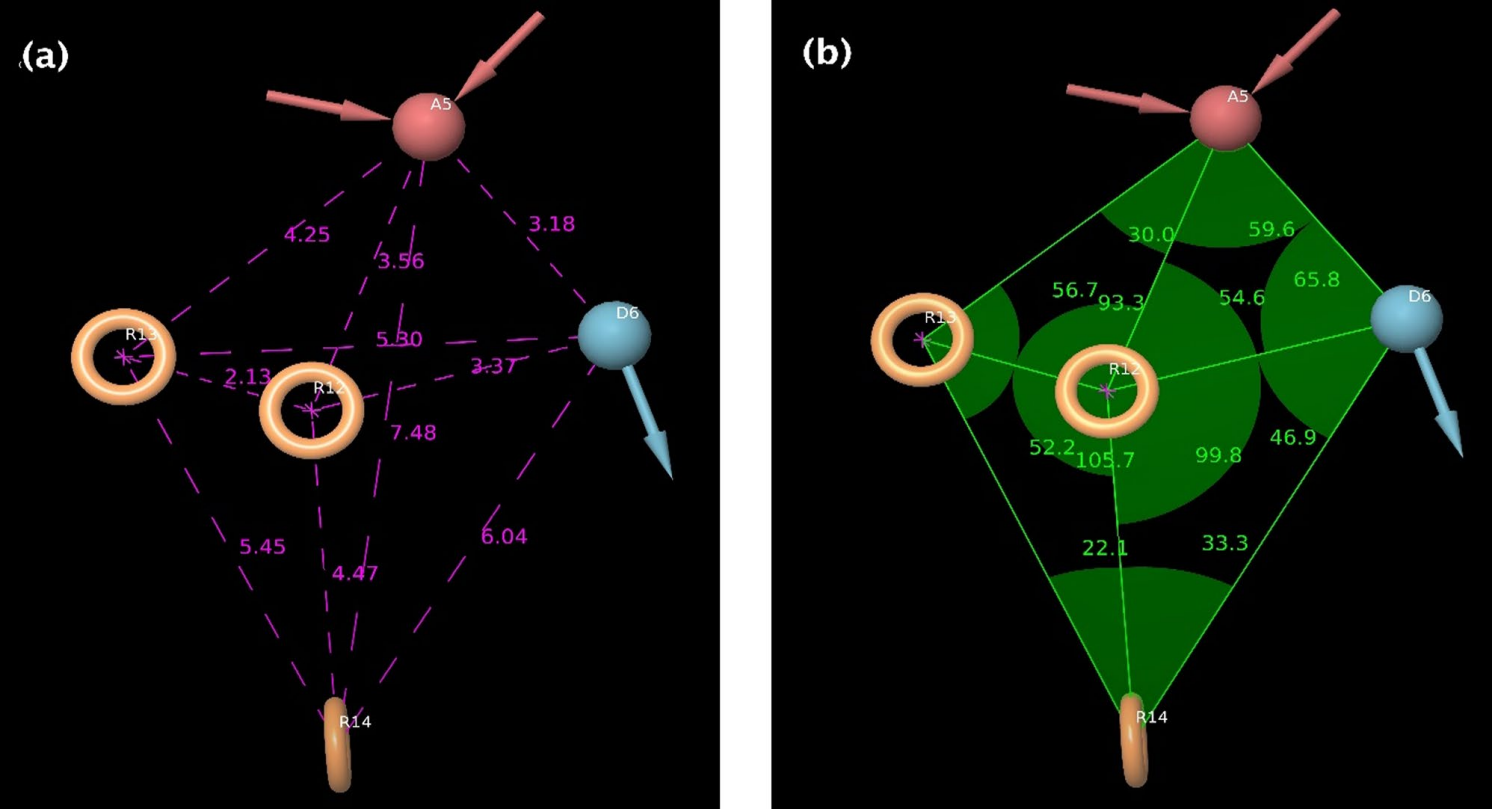
(**a**) The inter-site distance (Å) between the cubic sites of the ADRRR_1 model. (**b**) The angles (°) measured between different cubic sites of the ADRRR_1 model.

The top hypothesis ADRRR_1 has five pharmacophoric features comprising one H-bond acceptor (A5), one H-bond donor (D6), and three aromatic rings (R12, R13, and R14). These common pharmacophoric characteristics required for showing biological effects are visualized as an orange colour torus, pink colour sphere, and cyan colour sphere cubic sites, shown in Fig. 3c and d. As the 3D alignment of the generated pharmacophore hypotheses is essential, the inter-site distance and angles among the features of the picked pharmacophore model ADRRR_1 have been displayed in Fig. 5a,b and Table 3.

**Table 3:** Developed pharmacophore models in terms of their scoring parameters.

Distance between different pharmacophoric site points (Å)		Angles between different pharmacophoric site points (°)	
A5–D6	3.18	A5–D6–R12	65.8
A5–R13	4.25	A5–R13–R12	56.7
A5–R12	3.56	A5–R12–R13	93.3
A5–R14	7.48	R13–A5–R12	30.0
D6–R14	6.04	R12–A5–D6	59.6
D6–R12	3.37	A5–R12–D6	54.6
D6–R13	5.30	R12–D6–R14	46.9
R12–R13	2.13	R12–R14–D6	33.3
R12–R14	4.47	R14–R12–D6	99.8
R13–R14	5.45	R14–R13–R12	52.2
		R12–R14–R13	22.1
		R13–R12–R14	105.7

#### 3D-QSAR modelling analysis

40 DprE1 inhibitors with their pIC50 values (Supplementary Table S1) were selected for building a 3D QSAR model using the ‘Atom-Based QSAR’ option in the PHASE module. The 3D QSAR modeling describes the requisite structural characteristics of the inhibitors with increased and decreased actions needed for adequate binding with the receptor^41^. Initially, the dataset was split in the ratio of 70:30 as training and test sets with the application of partial least squares (PLS) as a regression method. The PLS method was mainly applied to analyze the robustness and the predictive ability of the developed 3D QSAR model through statistical calculations. Here, 4 nos of the PLS factors were used; among them, the PLS_4 factor showed the best regression statistics, as depicted in Table 4.

**Table 4:** Summary of statistical parameters of the generated 3D QSAR model.

Factors	SD	R^2^	R^2^ CV	R^2^ Scramble	Stability	F	RMSE	Q^2^	Pearson-r
PLS_1	0.6877	0.4358	0.6034	0.3475	0.861	20.1	0.41	0.5615	0.7747
PLS_2	0.4291	0.7887	0.5070	0.6068	0.338	46.7	0.42	0.5490	0.7459
PLS_3	0.2436	0.9347	0.8802	0.7261	0.195	114.4	0.32	0.7414	0.8700
PLS_4	0.1928	0.9608	0.9046	0.8229	0.172	140.8	0.32	0.7313	0.8563

*PLS* Partial Least Square, *SD* Standard Deviation, *R*^*2*^ Regression correlation coefficient, *R*^*2*^* CV* Cross validated R^2^, *F* Variance ratio, *RMSE* Root Mean Square Deviation, *Q*^*2*^ Correlation coefficient on the basis of external (test set) predictions.

**Figure 6:**
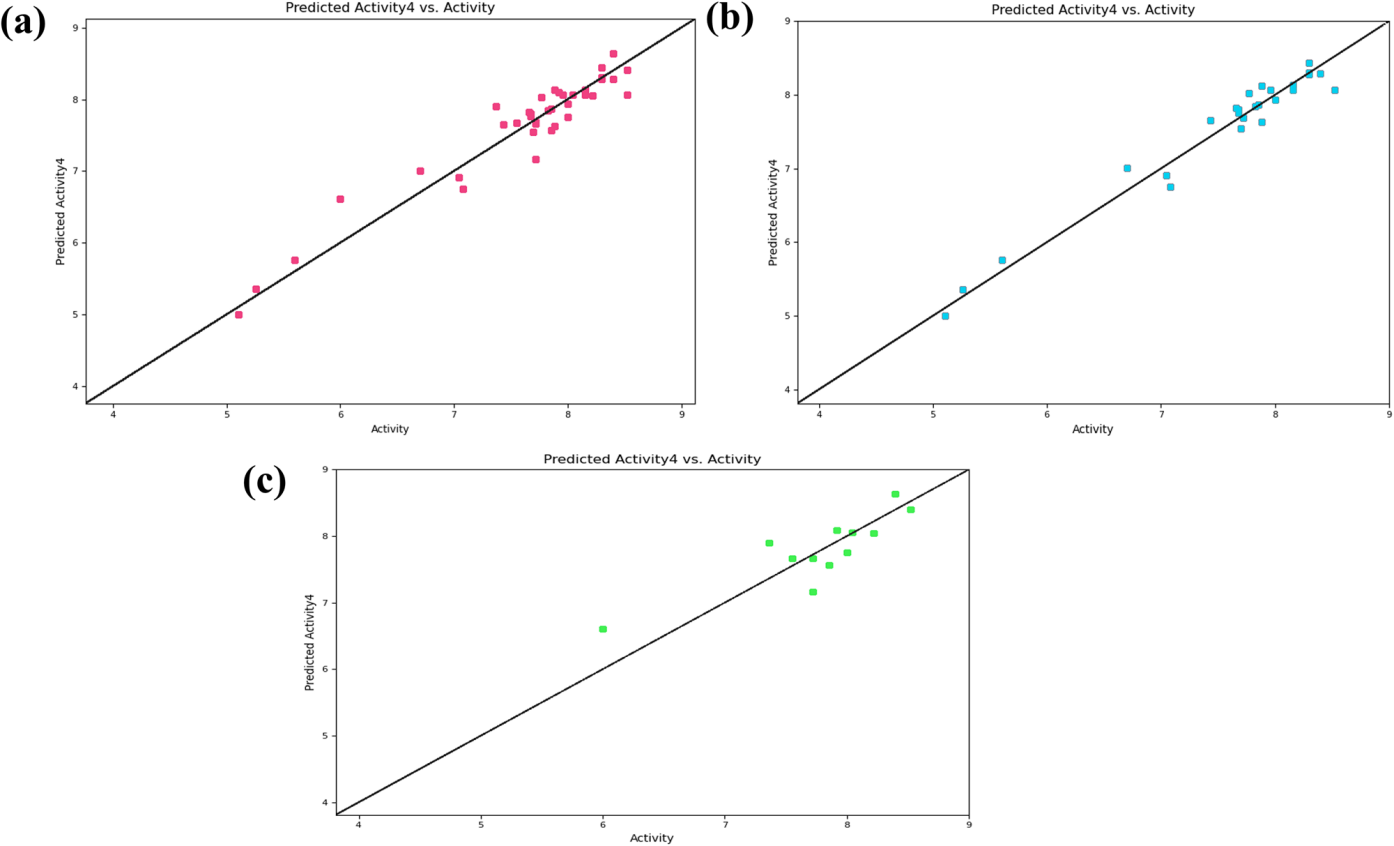
Linear scatter plots illustrating the correlation between the predicted activities and actual activities of (**a**) the used 40 dataset DprE1 inhibitors, (**b**) the training set, and (**c**) the test set generated by the 3D-QSAR model.

The validation of the constructed 3D-QSAR model was done internally by the value of R^2^ for the training set and externally by the value of Q^2^ for the test set. The built QSAR model displayed the significant values of R^2^ = 0.9608 and Q^2^ = 0.7313, indicating the good predicting capacity of the model. Further, the lower value of 0.19 for standard deviation and 0.32 for RMSE illustrated the best quality of the dataset for QSAR analysis. The more significant value of variance ratio (F = 140.8) and stability (0.172) revealed a degree of confidence. Also, the stability value of the model indicates the changes occurring in the composition of the training set. The Pearson-r value is mainly employed to validate the model externally, ranging from − 1 to + 1. Ideally, its value nearer to + 1 indicates a strong positive linear relationship. The value of 0.8563 for Pearson-r for this model represented a positive correlation. Furthermore, the correlation between the experimental and the predicted activities of the datasets showed less prediction error with a good correlation, as illustrated by scatter plots in Fig. 6. Also, the actual and predicted activities calculated by the built 3D-QSAR model for the training set and test set of the DprE1 dataset inhibitors are tabulated in Supplementary Table S1.

#### 3D-QSAR-based contour map interpretation

**Figure 7:**
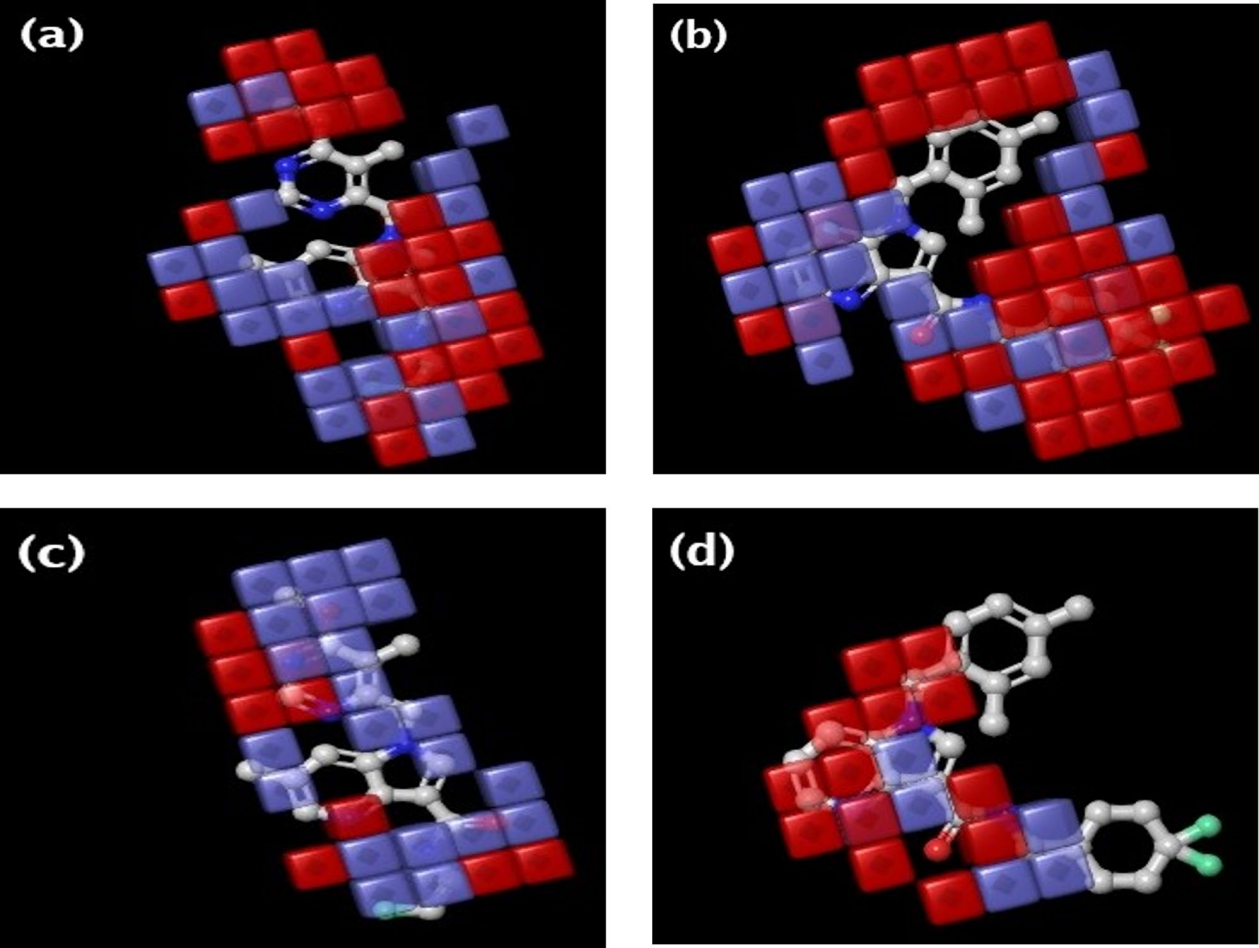
3D-QSAR based contour maps of hydrophobic effect for (**a**) most active compound **4** (**b**) least active compound **11**; electron-withdrawing effect for (**c**) most active compound **4** (**d**) least active compound **11** (where blue and red colour contours indicated the increased and decreased activity respectively.

The contour maps developed by the 3D QSAR-based model for both the most active compound **4**) and least active (compound **11**) DprE1 inhibitors assisted in visualizing the regions or groups showing the hydrophobic and electron-withdrawing effects. These contour maps also reveal the necessary structural features required for exhibiting biological activity. The favorable and unfavorable regions were indicated by blue and red colour contour cubes^42^. The contour maps showing the regions responsible for the hydrophobic effects and the electron-withdrawing effects for the most active molecule (Compound **4**) and the least active molecule (Compound **11**) are displayed in Fig. 7.

The red colour cubes over the pyrrole moiety of the primary azaindole scaffold of compound **4** (Fig. 7a) indicated its negative hydrophobic effects towards activity. Also, substituting the methoxy group on the pyrimidine ring attached to the N − 1 position of the azaindole showed negative results. This negative region was also demonstrated by compound **11**, which indicated that any further substitution would impact interactions and activity. In compounds **4** and **11**, the pyridine ring of the azaindole scaffold and the amide linker delivered positive hydrophobic effects toward action, indicated by blue contour cubes. However, the terminal 2-fluoroethyl group in compound **4** showed a positive effect, whereas the terminal 4,4-difluorocyclohexyl group showed a negative effect, which might be a reason for less activity (Fig. 7b). This suggested the incompatibility of the larger cyclic rings in this position.

The electron-withdrawing effects of the terminal 2-fluoroethyl group attached to the amide linkage might enhance the activity in compound **4**, which was surrounded by blue contours (Fig. 7c). Also, the positive contribution of the methoxy group toward electron-withdrawing effects assists in increasing its activity. However, the N-4 atom of the azaindole scaffold was displayed by red contours, indicating negative contributions. Similarly, both N − 1 and N − 4 atoms in azaindole showed negative electron-withdrawing effects in compound **11**, which might affect the activity (Fig. 7d). The detailed 3D-QSAR-based contour maps analysis will help design more potential DprE1 inhibitors with enhanced activity. Finally, the QSAR model developed in this study was utilized to predict the activities of the newly screened molecules along with the DprE1 clinical candidates for comparative evaluation purposes.

#### Pharmacophore hypothesis-based ligand screening results

A database of 23,99,743 compounds from chEMBL was preferred for database screening through the PHASE panel of Maestro. This study utilized the best pharmacophore ADRRR_1, like a 3D search query, to filter the hit compounds with similar pharmacophoric characteristics. These small molecules were primarily put in for ligand preparation to generate energy-minimized conformers or lowest-energy conformers. The chosen pharmacophore hypothesis ADRRR_1 was employed for ligand screening to identify the hit molecules. A total of 11,39,060 hit ligands were screened through this process, and the compounds with a phase screen score > 2 were picked for the next step of the molecular docking process^43^. The same hypothesis was also used to estimate the phase screen score of DprE1 inhibitors present in the clinical pipeline and co-crystallized ligand of (PDB ID: 4KW5) used for docking.

#### Ligand docking analysis of pharmacophore-screened hits

The obtained 15,257 hits with a phase screen score of > 2 were taken for HTVS docking by utilizing the prior generated receptor grid of (PDB ID: 4KW5). From HTVS docking, 2159 hits with docking score < − 7.0 kcal/mol were further selected for the SP method of docking to improve the accuracy of the accessed docking study. Again, 464 molecules with docking values < − 8.0 kcal/mol were subjected to the XP mode of docking to acquire the most accurate and reliable results by reducing the chances of false positives. Among these, 75 screened hits having docking values of lower than − 9.0 kcal/mol were picked for calculation of their free binding energies through Prime MM-GBSA. The docking results displayed the presumed binding interactions of the molecules in the substrate binding location of the selected receptor and exhibited the maximum docking values^44^. The co-crystallized ligand (TCA1) and clinical candidates targeting the DprE1enzyme were docked against the same protein structure (PDB: 4KW5) for comparative analysis. This comparative approach will enhance the interpretation and potential effectiveness of the identified compounds, which may present valuable insights into future anti-TB drug discovery. The docking values of these compounds were tabulated in Table 5. Along with this, the top-ranked (TOP 10) compounds showed docking scores within the − 9.701 to − 10.341 kcal/mol against the DprE1 target (PDB: 4KW5), depicted in Table 5.

**Table 5:** Docking scores (XP mode), binding free energies (dG bind), PHASE fitness scores, and 3D-QSAR predicted activities of top-ranked 10 hits, DprE1 clinical candidates and co-crystallized ligand (TCA1)**.**

Hit ID/compound name	Structure	Glide docking score (Kcal/mol)	Prime MM-GBSA (dG bind) (Kcal/mol)	PHASE fitness score	3D-QSAR predicted activity
Hit 1		− 10.341	− 66.74	2.028	7.537
Hit 2		− 10.302	− 61.45	2.023	8.279
Hit 3		− 10.215	− 53.06	2.033	7.882
Hit 4		− 10.189	− 41.28	2.122	7.805
Hit 5		− 10.129	− 42.40	2.033	6.564
Hit 6		− 10.049	− 43.76	2.028	8.274
Hit 7		− 9.928	− 43.95	2.015	7.315
Hit 8		− 9.906	− 42.18	2.050	6.706
Hit 9		− 9.904	− 47.81	2.053	6.802
Hit 10		− 9.701	− 49.46	2.054	8.021
BTZ-O43		− 4.975	− 48.84	ND	6.752
Mcozinone (PBTZ-169)		− 4.622	− 53.33	ND	6.862
TBA-7371		− 6.775	− 31.38	2.624	6.365
OPC-167832		− 6.468	− 30.21	1.305	6.868
Co-crystallized ligand (TCA1)		− 5.161	− 43.28	1.602	7.210

#### Free binding energies (Prime/MM-GBSA) calculations

The binding free energies of the 75 docked complexes were calculated by employing the Prime/MM-GBSA calculations. This Prime/MM-GBSA works like a rescoring function to illustrate the stability of the poses generated by the docked compounds. The calculated binding free energies were expressed as dG_bind,_ and the 39 hit molecules having dG_bind_ bind scores of < − 40.00 kcal/mol were considered for further lead optimization studies^17^. The free binding energies were also calculated for co-crystallized ligand (TCA1) and DprE1 inhibitors in clinical trials (Table 5). The top ten selected hits based on the highest docking scores with desired interactions, dG_bind_ values, PHASE screen scores, and QSAR-predicted activities were tabulated in Table 5.

*ND* Not determined.

#### Docking interactions profiling

**Figure 8:**
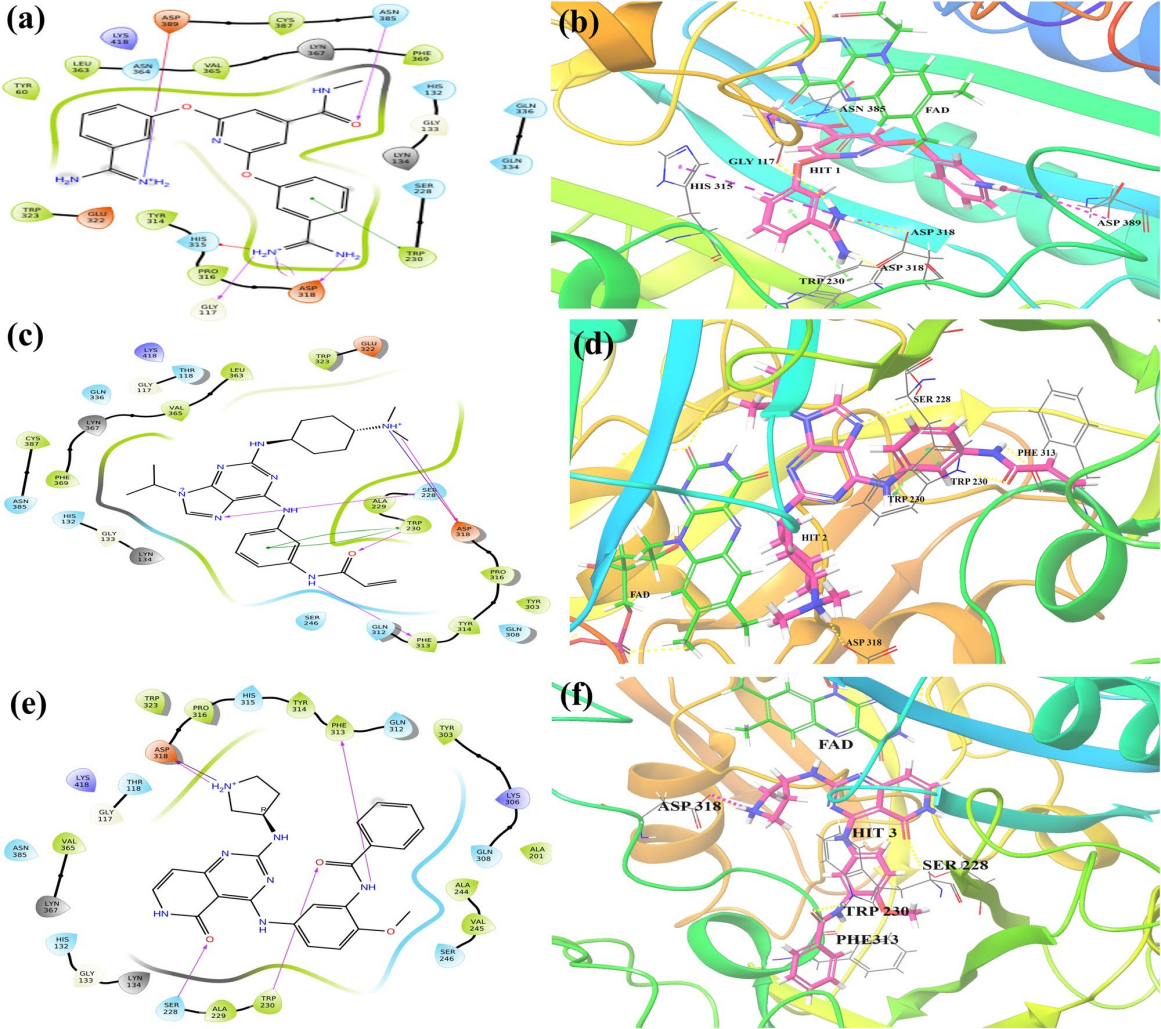
2D and 3D ligand interactions diagram of hit 1 (**a**, **b**), hit 2 (**c**, **d**), hit 3 (**e**, **f**) with DprE1 (PDB:4KW5).

**Figure 9:**
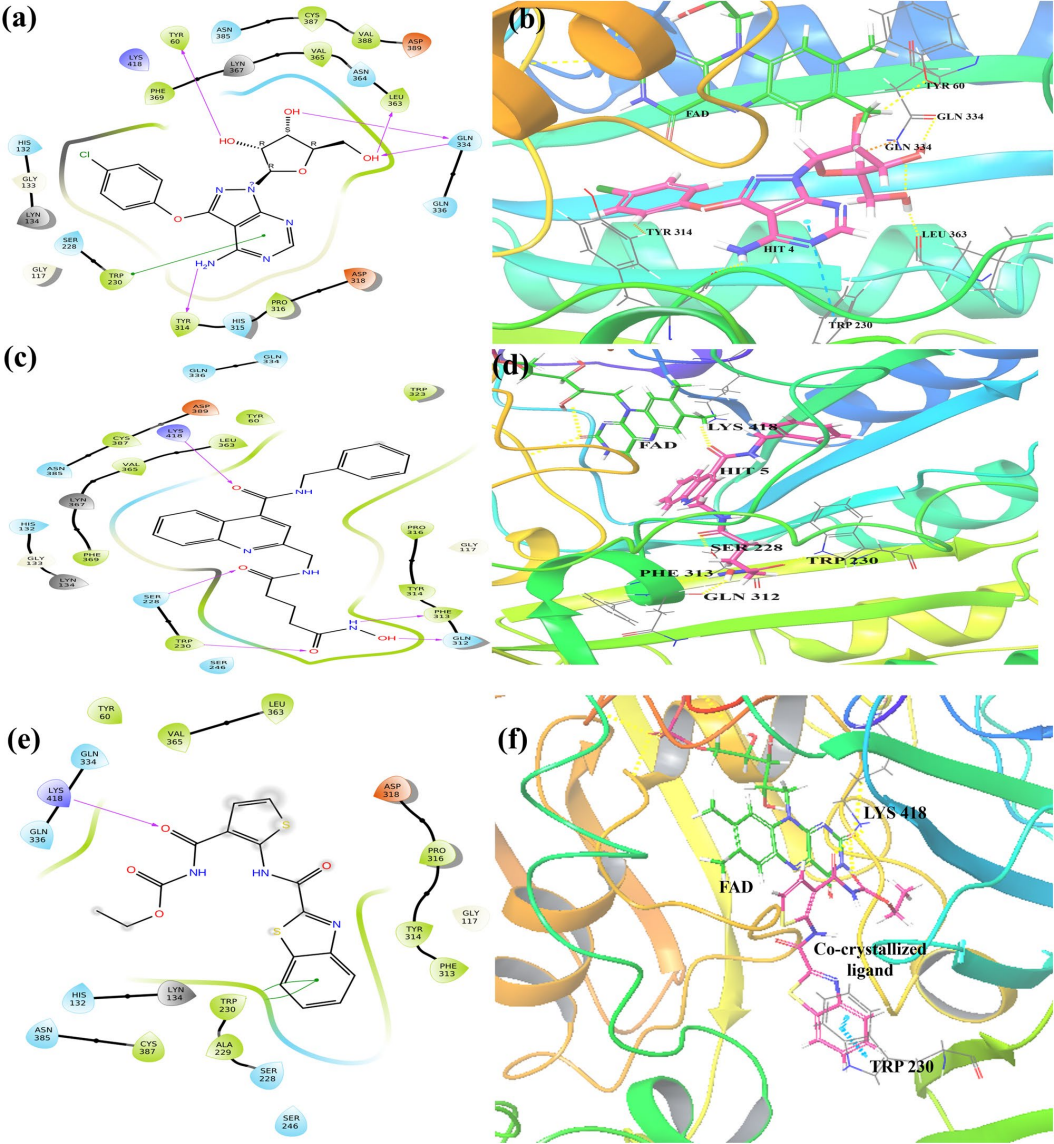
2D and 3D ligand interactions diagram of hit **4** (**a**, **b**), hit **5** (**c**, **d**), and co-crystallized ligand (**e**, **f**) with DprE1 (PDB:4KW5).

The top ranked (Hit **1–10**) molecules were suitably fitted into the DprE1 binding region and interacted with the desired amino acid residues via hydrophobic and hydrophilic bonding. The detailed mode of interactions obtained in XP docking methodology is demonstrated in Table 6. The 2D and 3D ligand interaction diagrams for the top five hit molecules (**1**, **2**, **3**, **4**, and **5**) and co-crystallized ligand (TCA1) are depicted in Figs. 8 and 9. And for hits (**6**, **7**, **8**, **9**, and **10**) and DprE1 clinical candidates (BTZ-043, macozinone/PBTZ-169, TBA-7371, and OPC-167832) the docking interaction diagrams are illustrated in Supplementary Fig. 2.

**Table 6:** Molecular docking interactions and IFD scores of top 10 hits.

Hit ID	Docking interactions	IFD scores
Hit 1	H-bond interactions: ASN 385, ASP 318, GLY 117 Salt bridges: ASP 389, ASP 318 Pi–Pi interactions: TRP 230 Pi-cat interactions: HIS 315	− 11.772
Hit 2	H-bond interactions: SER 228, TRP 230, PHE 313, ASP 318 Salt bridges: ASP 318 Pi–Pi interactions: TRP 230	− 10.411
Hit 3	H-bond interactions: SER 228, TRP 230, PHE 313, ASP 318 Salt bridges: ASP 318	− 10.506
Hit 4	H-bond interactions: TYR 60, TYR 314, GLN 334, LEU 363 Pi–Pi interactions: TRP 230	− 10.121
Hit 5	H-bond interactions: SER 228, TRP230, LYS 418, PHE 313, GLN 312	− 10.524
Hit 6	H-bond interactions: SER 228, TYR 314, GLY 117 Halogen bonding: GLN 336	− 10.378
Hit 7	H-bond interactions: TYR 60, ASP 318, GLN 334, LEU 363	− 10.131
Hit 8	H-bond interactions: SER 228, GLN 334 Pi–Pi interactions: TRP 230	− 10.144
Hit 9	H-bond interactions: ASN 385 PRO 316, VAL 365 Salt bridges: ASP 318 Pi–Pi interactions: TRP 230	− 11.088
Hit 10	H-bond interactions: SER 228, TYR 60, TYR 314, GLN 334, LEU 363 Pi–Pi interactions: TYR 314	− 9.142
BTZ-043	–	− 8.534
Mcozinone (PBTZ-169)	Salt bridges: ASP 318	− 8.905
TBA-7371	H-bond interactions: SER 228, TRP 230 Pi–Pi interactions: TYR 314	− 8.872
OPC-167832	H-bond interactions: TYR 60	− 10.013
Co-crystallized ligand (TCA1)	H-bond interactions: LYS 418 Pi–Pi interactions: TRP 230	− 7.880

The Hit **1** compound (chEMBL_SDF:11558) showed three hydrogen bond interactions with amino acid residues of ASN 385, ASP 318, and GLY 117 at the distances of 2.03 Å, 2.63 Å, and 2.67 Å, respectively. The receptor residues TRP 230 and HIS 315 showed a π-π interaction with the phenyl ring and a π–cation interaction with the NH atom of imine in the 2-(3-carbamimidoylphenoxy) moiety present in the compound. The –NH atom of imine in 2,6-bis(3-carbamimidoylphenoxy) also formed salt bridges with amino acids ASP 389 and ASP 318 (Fig. 8a,b).

The molecule hit **2** (chEMBL_SDF:357,100) exhibited four nos. of hydrogen bond (H-bond) interactions with SER 228, TRP 230, PHE 313, and ASP 318. The N7 atom of the purine nucleus formed an H-bond with SER228 at a distance of 2.47 Å. The -NH and the carbonyl group of the acrylamide moiety were bound with PHE 313 (2.44 Å) and TRP 230 (2.12 Å) residues of the target, respectively, through H-bonding. The amino acid ASP 318 was linked with the terminal dimethyl amine group of *N*,*N*-dimethylcyclohexanamine by forming a H-bond with a space length of 2.26 Å. Additionally, it was detected that one salt bridge also included between the terminal dimethyl amine group with ASP 318 and a π–π interaction shown by the phenyl ring of N-phenylacrylamide with TRP 230 of DprE1 (Fig. 8c,d).

Four hydrogen bonds were exhibited in Hit **3** (chEMBL_SDF:315738) in between 5-carbonyl oxygen group of pyrido[4,3-d]pyrimidin-5(6H)-one with SER 228 (2.12 Å) and carbonyl oxygen of N-(2-methoxyphenyl)benzamide linker with TRP 230 (1.98 Å). The other two H-bonds were observed in the –NH atom of N-(2-methoxyphenyl)benzamide linker with PHE 313 (2.03 Å) and –NH atom of terminal pyrrolidine moiety with ASP 318 (1.86 Å). An extra salt bridge was also formed among the NH atom of terminal pyrrolidine moiety and ASP 318 (Fig. 8e,f).

The hydroxymethyl group of 2-(hydroxymethyl)tetrahydrofuran-3,4-diol moiety in Hit **4** molecule formed two H-bonds with LEU 363 and GLN 334 at the distance of 1.77 Å and 2.78 Å. However, the hydroxyl group at the 3rd and 4th positions also showed hydrogen bond interactions with GLN 334 (1.93 Å) and TYR 60 (2.03 Å), respectively. The amine group in 1H-pyrazolo[3,4-d]pyrimidin-4-amine scaffold formed the other H-bond with TYR 314 at 1.99 Å of space distance. Like before hits, the pyrimidine ring in hit 4 also displayed a π-π interaction with TRP 230 (Fig. 9a,b).

The hit **5** molecule (chEMBL_SDF:314351) formed five hydrogen bonds with several amino acid residues such as LYS 418 (1.97 Å), SER 288 (1.93 Å), TRP 230 (1.80 Å), PHE 313 (1.73 Å), and GLN 312 (1.84 Å) of the DprE1 enzyme. The residue LYS 418 was connected with the carbonyl oxygen of 4-(benzylcarbamoyl)quinoline, whereas SER 228 was associated with the C5-carbonyl oxygen of hydroxyglutaramide moiety. The C1-carbonyl oxygen, –NH atom, and –OH atom shared hydrogen bonding with TRP230, PHE313, and GLN, respectively (Fig. 9c,d).

The hit **6** molecule (chEMBL_SDF:276439) exhibited three hydrogen bonds with the DprE1 enzyme. These were shown by the 1-OH group of 2-(hydroxymethyl)tetrahydro-2H-pyran-3,4,5-triol with GLY 117 (1.75 Å) and by 2,4-di-OH group with TYR 314 and SER 228 at the distance of 1.99 Å and 2.33 Å respectively. The –Cl atom in the 4-chloro-3-methoxybenzofuran ring also interacted with GLN 336 residue with 2.96 Å distance (Supplementary Fig. 2).

Like hit **5** molecule, hit **7** (chEMBL_SDF:233279) showed only H-bond interactions with residues of LEU 363 (2.75 Å), TYR 60 (1.85 Å), GLN 334 (1.94 Å), and ASP 318 (1.88 Å) of DprE1. These were shown by the –OH group present at the 2nd, 4th, 5th, and 6th positions of 1,2,4,5,6-pentahydroxyhexane connected with indazole scaffold through carboxylate linkage.

In the hit** 8** molecule, the carbonyl group at the 4th position of 2,4-dioxo-3,4-dihydroquinazoline scaffold displayed H-bond interactions with the desirable residue of SER 228 with a space distance of 1.94 Å. Two more H-bond interactions were shown by the carbonyl group and –OH group of N-hydroxybenzamide scaffold with GLN 334 with two different lengths of 2.04 Å and 1.98 Å, respectively. The phenyl ring of the 4-fluorophenethyl chain shared a π–π interaction with TRP 230 (Supplementary Fig. 2).

The hit **9** compound exhibited four H-bond interactions with the DprE1 residues. The terminal –OH group of propanol showed these hydrogen bonds with ASN 385 (2.09 Å) and VAL 365 (2.57 Å). The terminal –NH_2_ group of butane-1,4-diamine and N-atom of 6-(4-(morpholinomethyl)benzyl) group were associated with PRO 316 (2.53 Å) and TRP 230 (2.83 Å) respectively. This compound also displayed one π–π interaction with TRP 230 and salt bridges with ASP 318 like the prior hits (Supplementary Fig. 2).

The last selected hit molecule, hit **10**, showed six hydrogen bond interactions with different residues of amino acids. The 4-amino group of the primary scaffold, 1H-pyrazolo[3,4-d]pyrimidine bound with TYR 314 with a space distance of 1.83 Å. The –OH group in hydroxymethyl of 5-(hydroxymethyl)tetrahydrofuran-3,4-diol formed two H-bonds with LEU 363 (1.80 Å) and GLN 334 (1.81 Å). Also, the 3-OH group formed a H-bond with TYR 60 at a distance of 2.27 Å. The N-2 atom of (1-methyl-1H-pyrazol) attached at the 3rd position of 1H-pyrazolo[3,4-d]pyrimidine showed hydrogen bonding interactions with SER 228 (2.38 Å). Additionally, it formed one π–π interaction between 1-methyl-1H-pyrazol and TYR 314 (Supplementary Fig. 2).

The docking protocol was validated through a process involving the removal of the inhibitor from the complex, followed by re-docking. After re-docking, RMSD was calculated to be 1.5 which essentially verifies the accuracy and reliability of the docking protocol by comparing the spatial arrangement of the re-docked inhibitor with its original position in the complex (Supplementary Fig. 6). For the validation of docking analysis, we performed a comparative docking study of co-crystallized ligand (TCA1) bound with the protein (PDB:4KW5) and DprE1 inhibiting molecules currently reported as clinical candidates. Compared to the selected hits that showed docking scores in the range of − 9.7 to − 10.3 kcal/mol, the reported inhibitors showed docking scores in the range of − 4.6 to − 6.9 kcal/mol (Table 5). This suggests the better binding of the identified inhibitors with the DprE1 protein. TCA1 showed H-bond interaction with LYS 418 (2.05 Å) and a π–π interaction between the benzothiazole core with TRP 230 (Fig. 9e,f). BTZ-043 was ideally buried inside the pocket but did not show any interactions. In contrast, macozinone/PBTZ-169 shared only one salt bridge with ASP 318 (4.20 Å). Another clinical drug, TBA-7371, exhibited one π–π interaction between the 1,4-azaindole ring and TYR 314 (). The N4 atom of 1,4-azaindole scaffold and –OH group of N-(2-hydroxyethyl)-1H-pyrrolo[3,2-b]pyridine-3-carboxamide formed two H-bond with SER 228 (2.01 Å) and TRP 230 (1.91 Å), respectively. 3-hydroxy group of 3,4-dihydroxypiperidine moiety present in OPC-167832 displayed one hydrogen bonding interaction with TYR 60 (1.84 Å) (Supplementary Fig. 3). A comparative study was also performed to assess the free binding energy and predicted activity using the developed QQSAR model. As shown in Table 5, the identified hits showed comparable free binding energy profile and better predicted activity than the reported clinical inhibitors of DprE1.

#### Flexible docking (IFD) analysis

Based on the docking score, imperative interactions, binding energy score, and ADMET analysis results, the top 10 hit compounds were chosen for the IFD protocol. The IFD analysis of co-crystallized ligand and DprE1 molecules in the clinical phase was also carried out (Table 6). IFD mainly provides flexibility to both the small molecules and the target receptor during the docking method^45^. Up to 20 poses were obtained for each hit molecule, and the best pose for each hit was chosen on the basis of its IFD score for further molecular dynamics (MD) simulation procedure. The IFD scores of the top 10 hit molecules are displayed in Table 6.

#### Pharmacokinetics evaluation

The pharmacokinetic properties of all the 10 hit molecules were assessed to predict their lipophilicity, permeability, and druglikeness behaviors. All the hits fulfilled the metrics with some minor deviations. The molecular weights of all the hit molecules were within the concerned range of 130.0–725.0 Da and < 500 Da as per Lipinski’s rule of five. The number of hydrogen bond donors (not more than 6) and acceptors (between 2 and 20) for all the above molecules were in the acceptable range except Hit 1 (HBD: 7). Further, the hits also satisfied both the octanol/water partition coefficient (P_o/w_) and aqueous solubility (logS) parameters^46^. The prediction of IC_50_ values in the blockage of the hERG K^+^ channel in the form of QPlogHERG is one of the significant criteria because its blockage may develop QT prolongation, which leads to cardiac arrhythmias. The QPlogHERG values were within the range of − 4.457 to − 7.596. Permeability prediction to the Caco-2 cell line is calculated to study the in-vivo absorption for a drug as it represents the model-like gut-blood barrier. Hit 2, Hit 6, and Hit 8 showed the best PCaco values. All the hits were found to be CNS inactive and within the recommended range of blood–brain-barrier (BBB) permeability with QPlogBB values of − 0.769 to − 2.884. The amount of drug that reaches the target site can be predicted by calculating % human oral absorption. The results revealed Hit 2, Hit 6, and Hit 8 showed the highest percentage among the other hits^47^. ADMET properties of the selected hits from QikProp studies have been depicted in Table 7.

**Table 7:** ADME properties of top 10 hit molecules calculated by maestro QikProp module.

Hit ID	Mol wt.	HBD	HBA	QPlogPo_/w_	QPlogS	QPlogHERG	QPPCaco	QPlogBB	CNS	%Human oral absorption
Hit 1	404.427	7	7.5	1.454	− 3.733	− 6.259	27.119	− 2.884	− 2	48.153
Hit 2	462.597	3	9.0	3.860	− 5.715	− 7.249	234.399	− 0.769	− 2	91.967
Hit 3	471.518	4	9.2	2.538	− 4.667	− 7.596	42.430	− 1.510	− 2	70.939
Hit 4	393.786	5	10	0.312	− 3.482	− 5.634	68.266	− 1.977	− 2	61.599
Hit 5	420.467	4	10	0.861	− 3.198	− 4.457	35.797	− 2.471	− 2	59.797
Hit 6	464.899	4	10	2.170	− 4.124	− 5.191	285.697	− 1.418	− 2	83.610
Hit 7	485.320	5	11.5	1.731	− 3.976	− 5.863	62.993	− 2.346	− 2	69.282
Hit 8	433.438	2	7.7	3.464	− 5.347	− 6.511	245.552	− 1.466	− 2	90.009
Hit 9	454.574	5	10.9	1.341	− 1.757	− 7.086	14.603	− 1.298	− 2	55.640
Hit 10	347.333	5	11.8	− 0.889	− 2.831	− 4.932	44.985	− 2.120	− 2	38.368

*Mol. Wt.* Molecular weight (recommended: 130.0–725.0), *HBD* Hydrogen bond donors (recommended: 0.0–6.0), *HBA* Hydrogen bond acceptors (recommended: 2.0–20.0), *QPlogP*_*o/w*_ Octanol–Water partition coefficient (recommended: − 2.0 to 6.5), *QPlogS* Aqueous solubility parameter (recommended: -6.5—0.5), QPlogHERG: For blocking HERG K^+^ channel in terms of IC50 value (recommended: below − 5), *QPPCaco* Caco-2 cell line permeability (recommended: < 25: poor, > 500: great), *QPlogBB* Predicted Blood–Brain partition coefficient (− 3.0 to 1.2), *CNS* Central Nervous System activity prediction (recommended: − 2 (inactive), + 2 (active), *% Human oral absorption* Predicted human oral absorption (recommended: > 80% (high), < 25% (poor) ^46^.

The selected hits were also subjected to the computational prediction of their toxicity by the pkCSM web server tool for safe and successful drug discovery. AMES toxicity, maximum tolerated dose for humans, oral rat acute and chronic toxicity in LD_50,_ and LOAEL (Lowest Observed Adverse Effect) were evaluated for all the picked hit molecules^48^. Except for hit 8 and hit 9, the other compounds resulted in negative AMES toxicity that denoted their non-carcinogenic behaviors. The maximum tolerated dose (MTD) can estimate the toxic dose point of drugs or chemicals. MTD for all the hit compounds in this study was found to be in the acceptable range. Also, the LD_50_ and LOAEL values of the selected compounds demonstrated their low toxic nature^32^. The Insilco toxicity predicted values by the pkCSM tool have been tabulated in Table 8.

**Table 8:** In-silico toxicity profiles of top selected hits by pkCSM online tool.

Hit ID	AMES Toxicity	Max. tolerated dose (human) log (mg/kg/day)	Oral rat acute toxicity (LD_50_) mol/kg	Oral rat chronic toxicity (LOAEL) log mg/kg_bw/day
Hit 1	No	0.118	2.668	2.215
Hit 2	No	0.295	2.437	1.163
Hit 3	No	0.812	2.786	1.992
Hit 4	No	0.705	1.989	2.219
Hit 5	No	0.238	2.817	2.136
Hit 6	No	0.202	3.015	3.367
Hit 7	No	0.338	3.092	5.093
Hit 8	Yes	0.689	2.885	1.711
Hit 9	Yes	0.734	2.817	1.997
Hit 10	No	0.658	2.625	2.911

AMES Toxicity: No (Not mutagenic), Yes (mutagenic), Maximum tolerated dose (human): ≤ 0.477 (low), ≥ 0.477 (high), LD_50_: < 50 mg/kg (Toxic), ≥ 2000 mg/kg (non-toxic).

#### Druglikeness and synthetic feasibility estimation

The druglike properties of the selected molecules were evaluated by using five types of rule-based filters. These rule-established filters are mainly applied to find out the druglikeness of the compounds^35^. The results of the druglikeness calculation of the selected hits are provided in Table 9. As per the results, only hit 10 showed one violation in the Lipinski rule filter (molecular weight ≤ 500 Dalton, hydrogen bond acceptors ≤ 10, hydrogen bond donors ≤ 5, and ClogP (lipophilic nature) ≤ 5) due to more no of hydrogen bond acceptors. Five compounds (Hit **2**, **3**, **7**, **9**, **10**) demonstrated one violation in Ghose rule-based filter [-0.4 ≤ WlogP (lipophilicity) ≤ 5.6, 160 ≤ MW (molecular weight) ≤ 480, 40 ≤ MR (Molar Refractivity) ≤ 130, 20 ≤ noA (no. of atoms) ≤ 70]. According to Verber's rule of druglikeness (Total Polar Surface Area (TPSA) ≤ 140 & nos of rotatable bonds ≤ 10), some compounds exhibited violation for this. The compounds that showed violation in the Verber filter exhibited significantly less violation of more TPSA in the Egan rule-based filter [TPSA ≤ 131 & lipophilicity (logP) ≤ 5.88]. However, only hit **1** and **10** displayed one violation of TPSA > 150 as per Muegge’s filter [200 ≤ MW (molecular weight) ≤ 600, TPSA ≤ 150, − 2 ≤ logP (lipophilicity) ≤ 5, the no. of carbon atoms > 4, the no. of heteroatoms > 1, the no. of rings ≤ 7, hydrogen bond acceptors ≤ 10, hydrogen bond donors ≤ 5, the no. of rotatable bonds ≤ 15]^49^. In accordance with the above five rules, a compound can be active orally or absorbed orally if it does not break at least two of the preceding rules^50^. The results demonstrated that most of the hits fulfilled the above measure.

**Table 9:** Druglikeness estimation of the selected hits through the SwissADME web server.

SwissADME Filters	Hit 1	Hit 2	Hit 3	Hit 4	Hit 5	Hit 6	Hit 7	Hit 8	Hit 9	Hit 10
Lipinski violations	0	0	0	0	0	0	0	0	0	1 NorO > 10
Ghose violations	0	1 MR > 130	1 MR > 130	0	0	0	1 MW > 480	0	1 MR > 130	1 WLOGP < − 0.4
Verber violations	1 TPSA > 140	0	0	1 TPSA > 140	1 Rotors > 10	0	1 TPSA > 140	0	1 Rotors > 10	1 TPSA > 140
Egan violations	1 TPSA > 131.6	0	0	1 TPSA > 131.6	0	0	1 TPSA > 131.6	0	0	1 TPSA > 131.6
Muegge violations	1 TPSA > 150	0	0	0	0	0	0	0	0	1 TPSA > 150
Bioavailability	0.55	0.55	0.55	0.55	0.55	0.55	0.55	0.55	0.55	0.55
PAINS alerts	0	0	0	0	0	0	0	0	0	0
Brenk alerts	2 (imine)	1 (michael acceptor_1)	0	0	2 (hydroxamic acid, oxygen-nitrogen_single bond)	0	0	2 (hydroxamic acid, oxygen-nitrogen_single bond)	0	0
Leadlikeness	2 (MW > 350, Rotors > 7)	2 (MW > 350, Rotors > 7)	2 (MW > 350, Rotors > 7)	1 (MW > 350)	2 (MW > 350, Rotors > 7)	1 (MW > 350)	2 (MW > 350, Rotors > 7)	1 (MW > 350)	2 (MW > 350, Rotors > 7)	0
Synthetic accessibility	3.02	5.40	3.99	4.51	2.84	5.25	4.43	2.90	3.80	4.38

*NorO* No of hydrogen bond donors, *MR* Molar Refractivity, *MW* Molecular Weight, *WLOGP* Lipophilicity, *TPSA* Total Polar Surface Area.

The potentiality of a compound to circulate throughout the body can be measured by calculating its bioavailability score (0.1 to 1). The estimation of the bioavailability revealed that the same bioavailability score (0.55) was obtained in the case of all the studied hit molecules (Table 9), and the scores were also more than the ideal score values of > 0.25^51^. The compounds were also evaluated for structural alerts like PAINS (pan assay interference compounds) and Brenk, indicating the compounds' potent response in biological assay methods. In this study, no hit compounds showed PAINS alerts, but 2 Brenk alerts for hits **1**, **5**, **8**, and 1 Brenk alert for hit **2** were observed. According to a perfect definition of lead, a molecule will become suitable for future optimization, primarily by increasing size and lipophilicity. So, the leads must be smaller in size and less hydrophobic than druglike compounds^35^. However, except for hit **10**, all the hits showed lead-like violations because of higher molecular weight (MW > 350) and more rotatable bonds (Rotrors > 7). Additionally, the prediction of synthetic accessibility (SA) of all the selected molecules was determined to estimate the medicinal chemistry cordiality. The SA values were within the ranges from 1 (very easy) to 10 (very difficult) to synthesize. Overall, these preliminary data show the druglike and lead-like behaviors of the selected hits^52^.

#### Anti-TB sensitivity prediction assessment

Further, the hit compounds were investigated to predict their minimum inhibitory concentration (MIC) against eight diverse Mycobacterium species by employing an online mycoCSM server. Among them, only Mycobacterium tuberculosis (Mtb) MIC values were extracted and analyzed with marketed standards (Isoniazid and Rifampicin). The predicted Mtb MiC values calculated by mycoCSM are depicted in Table 10. The results found that the MIC values of the subjected hit molecules were close to the MIC value of isoniazid but lesser than that of rifampicin. Since a less MIC rating indicates, a lower amount of compounds is required to inhibit the growth phase of the organisms. Additionally, these results also justified our prior findings. Furthermore, in vitro and in vivo studies on Mtb are needed^53^.

**Table 10:** Anti-TB activity prediction of selected hits through machine learning online server mycoCSM.

Hits ID	Predicted Mtb. MIC (log µM)	Predicted Mtb. MIC (µM)
Hit 1	− 5.133	0.00589
Hit 2	− 4.861	0.00774
Hit 3	− 5.015	0.00663
Hit 4	− 4.471	0.01143
Hit 5	− 4.868	0.007688
Hit 6	− 4.401	0.01226
Hit 7	− 4.792	0.00829
Hit 8	− 4.716	0.00895
Hit 9	− 4.943	0.00713
Hit 10	− 4.263	0.01407
Isoniazid	− 4.942	0.00714
Rifampicin	− 6.13	0.00217

#### Molecular dynamics simulation (MDS) result analysis

The primary goal of performing MD simulations was to verify the stability of the DprE1-ligand complex in physiological environments, which could not be achieved via IFD or ligand docking. Depending on prior scores of anti-TB sensitivity, ligand docking, and ADMET/druglikeness studies, five hit compounds (Hit **1**, **2**, **3**, **5**, and **9**) were selected with the DprE1 enzyme for MD simulations analysis. Initially, a 200 ns period of MD simulations with 1000 frames was run by taking the best IFD poses of hit molecules. The stability of the hits with the DprE1 binding site was estimated by evaluating the plots of RMSD (Root Mean Square Deviation), RMSF (Root Mean Square Fluctuations) of both protein and ligand, and also interactions of both ligand and protein.

**Figure 10:**
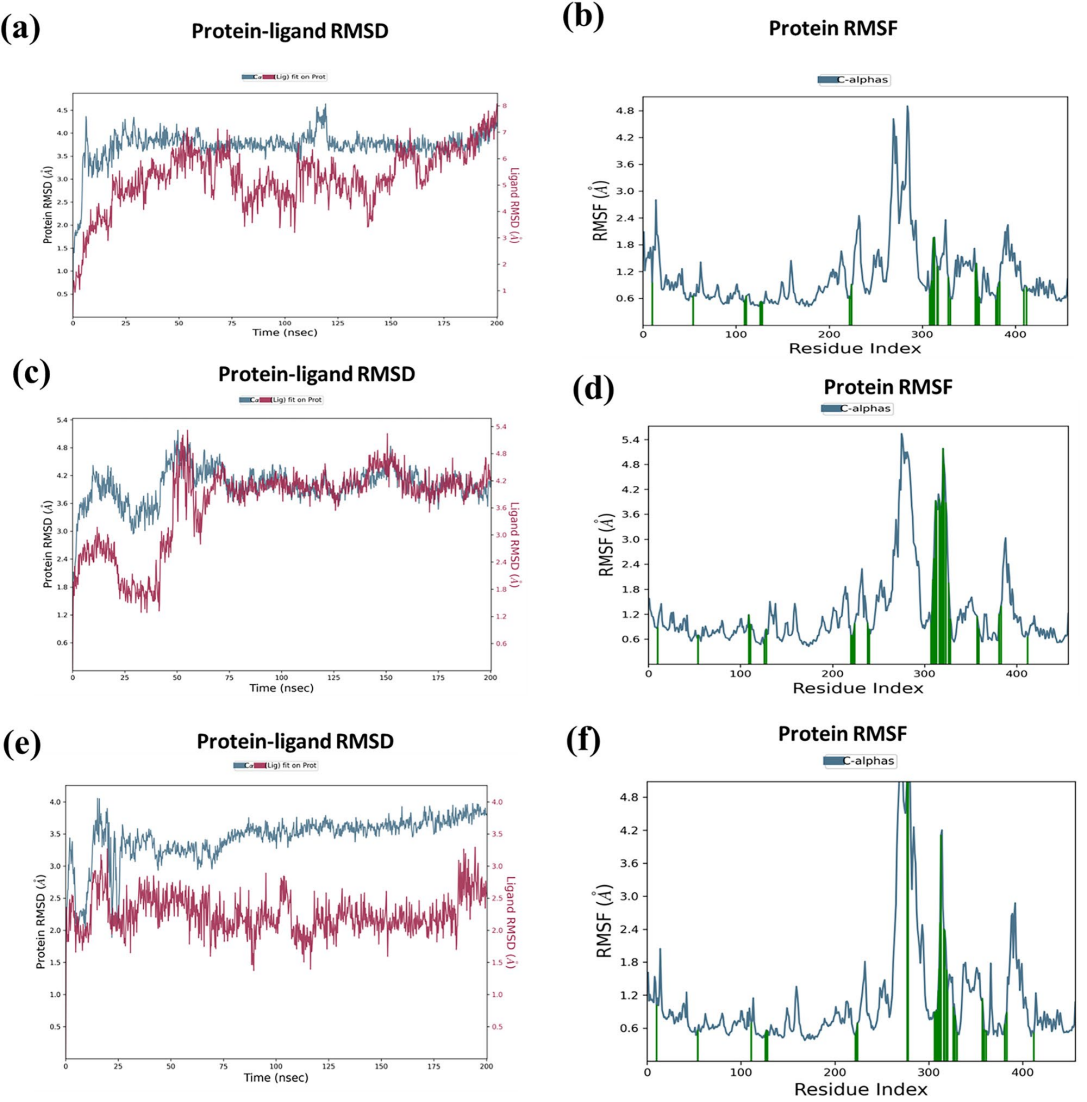
DprE1 protein–ligand RMSD plot of (**a**) hit 1 (**c**) hit 2 (**e**) hit 3 and RMSF plot of DprE1 with (**b**) hit 1 (**d**) hit 2 (**f**) hit 3.

RMSD plot could provide information about the variations in conformations of protein concerning ligands^40^. The complex of DprE1-hit **1** was unstable from the beginning up to 50 ns and became stable from 50 to 75 ns; again, it became unstable till 150 ns, after which, for a 150–160 ns period, it showed stability, then a slight drift was shown, and from 175 ns, it stabilized till the end (Fig. 10a). In the complex of DprE1-hit **2**, unsteady conformations were observed till 50 ns, then a short period of stable state from 50 to 60 ns appeared; after a slight fluctuation from 60 to 75 ns, it became stable throughout the simulations, as displayed in Fig. 10c. After partial stability from the initial 0–25 ns period, the complex hit **3**-DprE1 showed fully unsteady conformations until the end of 200 ns simulations (Fig. 10e). Previously becoming unstable till 75 ns, the complex of hit **5** became stable for a short term till 120 ns, but again, minor deviations in conformations up to 200 ns occurred (Supplementary Fig. 4a). In the DprE1-hit **9** complex, stability was observed in the initial phase of simulations till 60 ns, then became unstable throughout the simulation period of MD. The average RMSD results indicated the highest stability of hit 2 complex throughout simulations (Supplementary Fig. 4c).

The RMSF plot depicted the fluctuations that occur around the protein chain in the presence of ligands^26^. The RMSF values ranging from 0.6 to 5.4 Å were obtained from the simulation results. It was noticed that fewer fluctuations occurred in the DprE1 protein chain when it was bound with hits **1** (Fig. 10**b**) and **2** (Fig. 10**d**), but high fluctuations were observed in the case of hits **3**, **5**, and **9** complexes with DprE1. Figure 10f depicts the conformational changes occurring in the DprE1 side chain during the binding of hit **3**. The RMSF plot of the DprE1 protein in the presence of hits **5** and **9** are demonstrated in Supplementary Fig. 4b and d, respectively.

The histogram illustrates the protein contacts with the ligand, which indicates the duration of the period in percentage till which the particular interaction is retained. A score of 0.5 denotes that the specific contact is prolonged till 50% of the simulation period. In contrast, a score of more than 1.0 means the formation of extra connections with the ligand by that same protein residue^40^. This histogram also represents the different kinds of bonds formed between the hit molecules, the crucial residues of DprE1, and their stability during the 200 ns simulations.

Hit 1 resulted in H-bonding interactions with ASP 389, HIS 315, LEU 363, SER 228, GLN 312, and PHE 313 with interaction fractions of 1.55, 0.65, 0.50, 0.20, 0.35, and 0.40, respectively.

**Figure 11:**
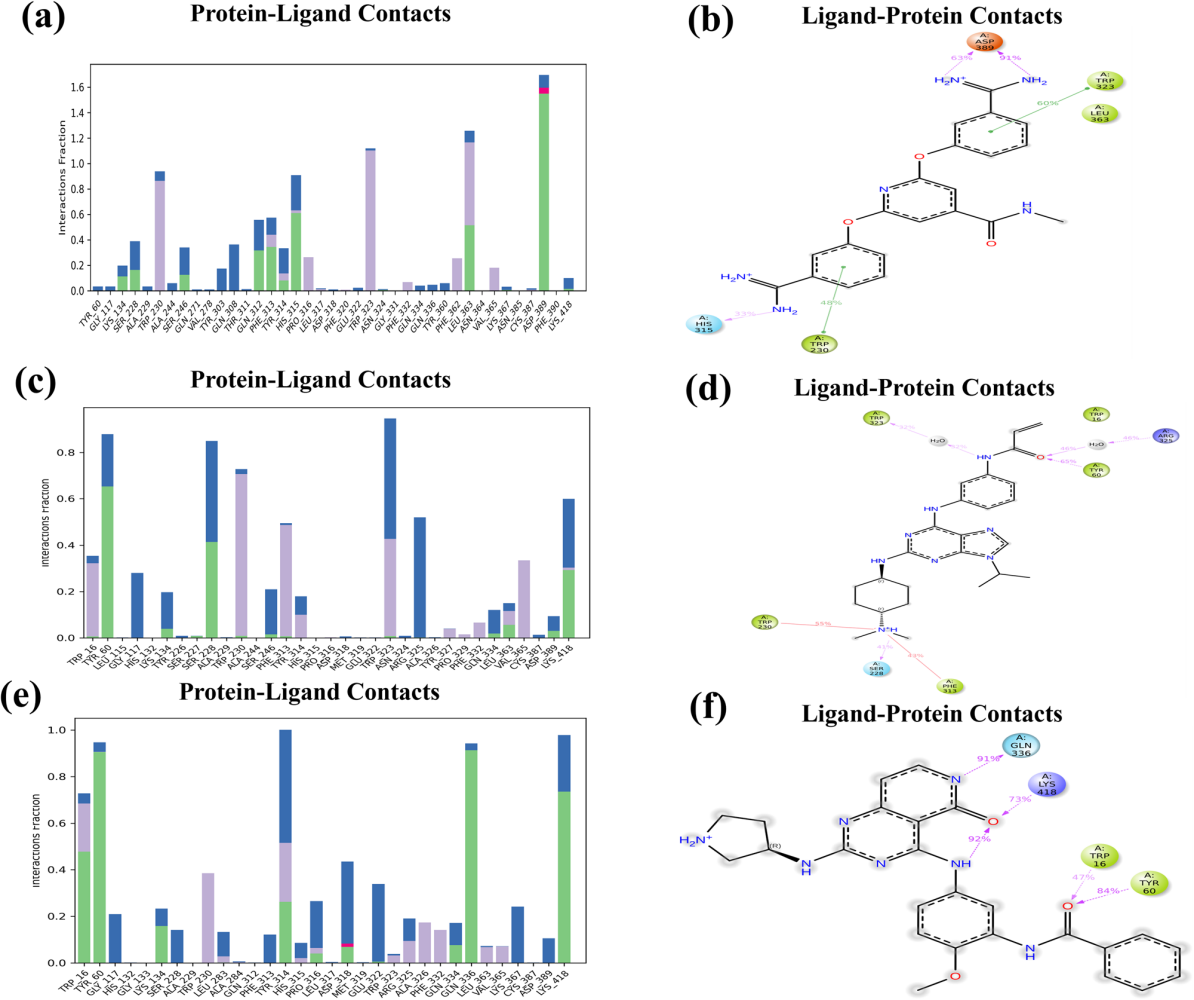
DprE1-ligand contact histogram (**a**) hit 1 (**c**) hit 2 (**e**) hit 3 and interaction diagram of (**b**) hit 1 (**d**) hit 2 (**f**) hit 3 with DprE1 residues.

The residues of TRP 230 (0.85), PRO 316 (0.30), PHE 362 (0.32), and TRP 323 (1.17) participated in forming hydrophobic interactions with DprE1 (Fig. 11a). A total no of three hydrogen bonds with TYR 60 (0.65), SER 228 (0.41), and LYS 418 (0.37) were shown by hit **2** during 200 ns simulation time. The receptor residues of TRP 16, TRP 230, TRP 323, PHE 313, and Val 365 had hydrophobic interaction scores of 0.37, 0.70, 0.42, 0.50, and 0.35, respectively (Fig. 11c).

As the RMSD plot of hit 3 was not consistent till the end of the 200 ns simulations, still five no of stable hydrogen bonds with TRP 16 (0.43), TYR 60 (0.90), TYR 314 (0.25) GLN 336 (0.90), and LYS 418 (0.75) were observed. Additionally, the interaction scores of 0.40, 0.21, 0.16, 0.05, and 0.04 were shown by TPR 230, ALA326, PHE 332, LEU 363, and VAL 365 through weak hydrophobic interactions in the histogram of protein–ligand (hit 3) contacts (Fig. 11e). In the complex of hit **5**-DprE1, the interaction values of 0.03, 0.05, 0.18, 0.43, 0.80, and 0.17 were shown by hydrogen bond forming amino acids of TYR 60, GLY 117, THR 118, ASP 318, ASP 389, and LYS 418 during MD simulations. Approximately ten no of hydrophobic contacts with TRP 230 (0.35), LEU 275 (0.20), PHE 279 (0.18), TYR 314 (0.57), PRO 316 (0.19), TRP 323 (0.6), PHE 332 (0.32), PHE 362 (0.22), VAL 365 (0.10) was also formed by hit **5** molecule (Supplementary Fig. 5a). During the 200 ns simulation time of DprE1- hit **9** complex, the interaction scores were 0.92, 0.45, 0.16, 0.10, and 0.37 for DprE1 residues of SER 228, ASP 318, LEU 363, ASN 385, and ASP 389, respectively through hydrogen bonding. Two hydrophobic contacts were also observed with TRP 230 (0.42) and VAL 365 (0.35) by hit **9** molecule (Supplementary Fig. 5c). In general, hit 2 displayed an extended contact period than the other selected hit molecules.

The report of contacts between ligand–protein demonstrated the interactions retained in the maximum percentage (> 30%) of simulation time^54^. The protein–ligand interactions, bonding categories, and residue interactions were observed as a bar graph of the histogram. The significant residues ASP 389 and HIS 315 maintained charged (negative) and polar interactions with hit **1** up to simulation time of 91% and 33%, respectively. Whereas hit **1** also kept its hydrophobic (π–π stacking) contacts with TRP 230 (48%) and TRP 323 (60%) in a 200 ns simulation period (Fig. 11**b**). During the period of simulation of the DprE1-hit **2** complex, TYR 60, TRP 323, TRP 230, and PHE 313 residues retained their hydrophobic interactions with the % simulation time of 60%, 32%, 55%, and 43%, respectively. Along with these interactions, hit 2 also formed polar and charged (positive) contacts with SER 228 and ARG 315 of DprE1 up to 41% and 46% of simulation durations (Fig. 11**d**). Hit **3** molecule showed charged (positive) contacts with LYS 418 (73%), polar contact with GLN 336 (91%), and two hydrophobic contacts with TRP 16 (47%) and TYR 60 (84%). It also formed an intramolecular hydrogen bond with 92% of simulations, as shown in Fig. 11f. In the complex of hit **5** with DprE1, two charged (negative) contacts with ASP 389 and ASP 318 and one hydrophobic (π-π stacking) contact with TYR 314 were observed at 71%, 37%, and 35% of simulation time, respectively (Supplementary Fig. 5b). The last selected hit **9** maintained two polar contacts with SER 228 and GLN 336 and one hydrophobic (π–π stacking) contact with TYR 314 up to 89%, 39%, and 34%, respectively. Additionally, it also exhibited two negatively charged and one positively charged contact with ASP 318 (46%), ASP 389 (31%), and LYS 418 (69%) during the entire simulation period (Supplementary Fig. 5d). Therefore, as per the complete reports of the MD analysis, hit 2 demonstrated a strong binding affinity and consistent stability with the DprE1 protein, which may be used as a promising molecule in future TB drug development.

## Discussion

The enduring resistance crisis to the current anti-TB drugs is a matter of dreadful concern in the Global TB control program. Also, the extended duration of TB treatment, adverse actions of existing anti-TB medications, and restricted efficacy of drugs toward resistant forms of TB upsurge the mortality rate in TB patients by making the treatment fail^55^. These conditions have engaged scientists worldwide in developing novel potential drug molecules. As profound research is occurring on new TB drug discovery and development, more studies are still taking place to determine the new molecular lead with better potency and less toxicity. Among those, extensive researchers have been accomplishing their studies on DprE1 by developing diversified scaffolds. Mtb-DprE1 and its isoform, Mtb-DprE2, are the two foremost units in arabinogalactan (AG) and lipoarabinomannan (LAM) synthesis. These two enzymes mainly engage in forming the sugar component, d-arabinofuranose (Araf), through synthesizing its precursor, Decaprenylphosphoryl arabinose (DPA), by epimerization method^8^. As the in-silico techniques have become relevant in the drug identification process, we performed our studies by merging the DprE1 inhibiting ligand-based and DprE1 structure-based virtual screening techniques. Shirude et al. reported an azaindole scaffold containing DprE1 selective inhibitors, and among these, TBA-7371 (Compound 5, Supplementary Table S1) is in the clinical phase. In this current work, we chose these reported azaindole-based DprE1 inhibitors for 3D-QSAR and pharmacophore modeling in the quest for the new DprE1 inhibitors. Several in-silico studies were conducted on DprE1 inhibitors by employing DprE1 enzymatic structure through structure-based pharmacophore modeling or direct ligand docking. Similarly, we used a ligand-based approach to perform our studies of identifying new DprE1 inhibitors. Although 20 divergent pharmacophore hypotheses were developed, the best ADRRR_1 hypothesis was chosen by reviewing their enrichment results and other parameters like survival score (5.484), phase score (1.327), etc. In the selected five-point pharmacophore frame, ADRRR_1, A denoted H-bond acceptor, D denoted H-bond donor, and R characterized aromatic site required for the alignment of molecule set targeting DprE1. Later, the above pharmacophore model was used for the chEMBL database (23,99,743 molecules) screening, and this method was accomplished by filtering 15,257 molecules with phase scores > 2 and 4 out of 5 pharmacophore matching features.

**Figure 12:**
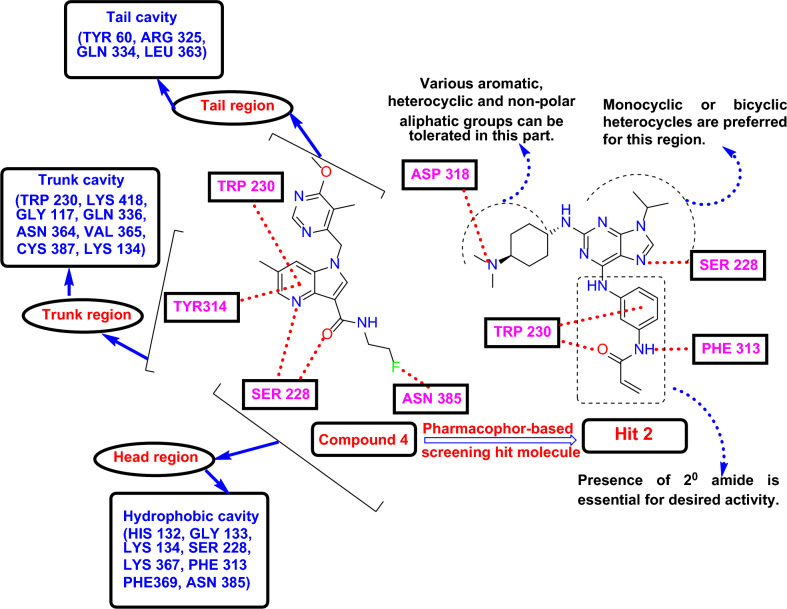
Brief SAR associated with interactions study of compound 4 and hit molecules.

Further, the study of DprE1 structure-based virtual screening was carried out with screened molecules-DprE1 structure docking through different modes of docking (HTVS-SP-XP) by utilizing the receptor grid of the minimized protein (PDB: 4KW5). The filtered 75 molecules with XP dock score < − 9.0 kcal/mole were dominated for the following MM-GBSA calculations. From the MM-GBSA analysis, the screened hits (39 molecules) were selected with dG_bind_ bind scores of < − 40.00 kcal/mol. The evaluation of the ADME/T parameters was further demonstrated, resulting in 10 best hits. The DprE1 inhibition actions of the final 10 hit molecules were predicted employing the built QSAR model. The interpretation of QSAR statistical parameters (R^2^ = 0.9608, Q^2^ = 0.7313, and Pearson-r = 0.8563) indicated the significant predictive power of the built model. The critical information on SAR is a foundation in drug discovery methods, which guide the selection and optimization of the best compounds by determining the relationship between a chemical moiety's structure and its actions. Here is an outline of the brief SAR investigations of the top hits and their interactions with DprE1 residues. By resembling the structural characteristics and interactions of the hit molecules with the reported most active compound (compound **4**), the summary of this SAR analysis is depicted in Fig. 12.

The SAR exploration in this work has relied chiefly on the information provided by the recently published review on the active site areas of DprE1^56^. As per the details mentioned, the head portion is accountable for the drug binding and inhibitory actions, and this portion is occupied by the hydrophobic cavity of residues, ASN 385, SER 228, LYS 367, PHE369, LYS134, GLY 133, and HIS132. This SAR analysis suggests that H-bond (non-covalent) interaction with SER 228 may enhance DprE1 inhibitory activity by forming a strong interaction. It is distinguished that the 2^0^ amides bound with these desired residues are preferred for showing actions. The pockets of residues such as GLY 117, LYS 418, CYS 387, VAL 365, ASN 364, TRP 230, and LYS 134 covered another trunk part of the active site, which mainly accommodated the monocyclic and bicyclic rings of heterocyclic molecules inside it. The π–π interaction with TRP 230 and the extra H-bond with LYS 418 extended the stability of the molecules. It was observed that most of the selected exhibited π–π interaction with TRP 230. The tail section is the solvent-approachable region where the aromatic and heterocyclic groups can be abided. The amino acid TYR 60 is significant for binding molecules and other residues like LEU 363, GLN 334, and ARG 325. Therefore, the hydroxyl group-containing aliphatic chain and cyclic moiety of the hit molecules showed interactions with these residues. It was also observed that all the selected hits were perfectly bound in the binding pocket and showed interactions with the above amino acid residues. Also, all the screened hits (HIT 1 to 10) showed better docking scores and binding interactions than co-crystallized compound and DprE1 clinical molecules. Overall, this indicates the robustness of the computational approaches employed in this study's findings. A straightforward elucidation of the SAR analysis might assist in the future discovery of DprE1-inhibiting ligands by providing ideas on the fundamental structural features necessary for showing activities.

The binding free energies generally demonstrate the stability of the docking poses. In this study, we preferred the molecules with dG_bind_ bind scores of < − 40.00 kcal/mol by using the dG_bind_ bind score of the co-crystallized ligand (− 43.28 kcal/mol). Despite showing higher docking scores and absence of binding interactions, two clinical compounds, BTZ-043 (− 48.84) and macozinone/PBTZ-169 (− 53.33), showed lower binding energies than TBA-7371 (− 31.38) and OPC-167832 (− 30.21). This indicates the stability of these two compounds in the DprE1 receptor. These selected hits' toxicity and druglikeness profiles were evaluated using freely available online servers. The values shown by all the hits are within the parameter with some minor deviations. Among them, hit **2** exhibited the highest % of human oral absorption score (91.967%). The prediction of the anti-TB sensitivity of these chosen hit molecules and the standards, isoniazid and rifampicin, was also interpreted. The values of the hits are nearer to the value of isoniazid but less than rifampicin.

Moreover, flexible docking analyses, such as IFD and MD simulations, were accomplished to illustrate complex stability. The interpretation of the multiple poses generated by the complex is the primary purpose of doing IFD, from where the best poses of molecules are taken for subsequent simulation analysis. However, none of the above-performed studies can imitate the body receptor–ligand environment, so 200 ns MD simulations are carried out for the top five small molecules. The results of MD demonstrated better DprE1-hits interactions with good RMSD, RMSF, and ligand-contacts plots. The outcome of the current study could contribute new insights to the researchers for developing potential DprE1 inhibitors.

While our study contributes valuable insights into identifying potential DprE1 inhibitors, it is important to acknowledge some limitations that may have impacted the interpretation and generalizability of our findings. These limitations include the lack of experimental validation of our findings in an in vitro or in vivo assay setup, and the dataset for QSAR modeling and pharmacophore screening could have been larger and more diverse, resulting in a better QSAR model.

## Conclusion

Tuberculosis is usually regarded as one of the deadliest infections in humans that triggers worldwide mobility and morbidity due to existing drug resistance. This highlights the requisite of TB treatment by novel anti-TB agents that can bring down the global burden of TB. The significance of the Mtb cell envelope in the pathogenesis of TB makes this a historical target, so the impediment to its process of biosynthesis has been a cornerstone of TB drug development. DprE1, a ruling enzyme in the formation of arabinogalactan (AG) and lipoarabinomannan (LAM), has become a research aim for many scientists^8^. Our research has centered on developing potential molecules against DprE1 through virtual screening by integrating both ligand structure and target structure-based drug discovery techniques. In this conducted study, we designed a similar scaffold bearing a multiple ligand-based pharmacophore model and a 3D-QSAR model using 40 azaindoles of DprE1 inhibitors. The chEMBL database was screened virtually using the best pharmacophore hypothesis (ADRRR_1). Further, structure-based screening was carried out for the filtered compounds using the DprE1 structure (PDB: 4KW5). Based on the docking values (XP docking score < 9.0 kcal/mol), the resulting top hits were nominated for calculating MM-GBSA free binding energies. The docked molecules also showed the desired interactions with the DprE1 residues of Tyr 60, Ser 228, Trp 230, Tyr 314, Val 365, Asn 385, and Lys 418. The compounds with dG bind scores < − 40.00 kcal/mol were further considered for ADME evaluation. The compounds' toxicity and druglikeness parameters were also calculated to determine safe lead molecules. This resulted in the selected molecules with acceptable ADME/T and druglikeness characteristics. The top 10 best hits obtained from these screening procedures were further picked for IFD analysis and for prediction of anti-TB sensitivity. The generated 3D-QSAR model also predicted the activity of DprE1 inhibition by the selected molecules. The MD simulations were also performed for the top five hits to analyze stability. The simulation results showed stable interactions of hits with DprE1 protein during 200 ns simulations with good RMSD, protein, and ligand RMSF, protein–ligand, and ligand–protein contacts. By validating all the results, hit **2** could work as a new inhibitor of DprE1 for treating tuberculosis, and this study could assist in developing potent DprE1 inhibitors in the future.

### Supplementary Information

Supplementary Information.

## Data Availability

The datasets used in the manuscript are publicly available from the repositories below: (1) Repository Name: RCSB Protein Data Bank; Deposited Date: 2013-05-23; Released Date: 2013-07-10; by source author(s): Batt, S.M., Besra, G.S., Futter, K. Accession Number: 10.2210/pdb4KW5/pdb; Macromolecular structure:4KW5[link to the repository: https://www.rcsb.org/structure/4KW5] and originally deposited from article, 10.1073/pnas.1309171110. and (2) Repository Name: chEMBL database [link to the repository: https://www.ebi.ac.uk/chembl/g/#search_results/all] and (3) other datasets generated during the current study can be made available from the corresponding author upon reasonable request.
