# Supplementary material for: Identification of DprE1 inhibitors for tuberculosis through integrated in-silico approaches
Source: Sci Rep. 2024 May 17;14:11315. doi: 10.1038/s41598-024-61901-x (PMC11101490; doi:10.1038/s41598-024-61901-x)
Supplement: Supplementary file 1 — Supplementary Information. [file 41598_2024_61901_MOESM1_ESM.docx]

Supplementary Material

**Identification of DprE1 Inhibitors for Tuberculosis Through Integrated *In-silico* Approachs**

**Swagatika Dash^1^, Ekta Rathi^1^, Avinash Kumar^1^, Kiran Chawla^3^, Suvarna G. Kini^1, 2^***

**^1^ Department of Pharmaceutical Chemistry, Manipal College of Pharmaceutical Sciences, Manipal Academy of Higher Education, Manipal, Karnataka, India-576104**

**^2^Manipal Mc Gill Centre for Infectious Diseases, Prasanna School of Public Health, Manipal Academy of Higher Education, Manipal, Karnataka, India-576104**

**^3^Department of Microbiology, Kasturba Medical College, Manipal Academy of Higher Education, Manipal, Karnataka, India-576104**

**Corresponding author-**

**Dr. Suvarna G Kini**

**M. Pharm, PhD**

**Professor and Head, Department of Pharmaceutical Chemistry, Manipal College of Pharmaceutical Sciences, Manipal Academy of Higher Education, Manipal, Karnataka, India-576104**

**Email id-** [suvarna.gk@manipal.edu](mailto:suvarna.gk@manipal.edu)

**Telephone no- +91-9880713601**

**Contents**

Supplementary Table S1: Structures and their reported biological activities (IC_50_) of 39 azaindole scaffold based DprE1 inhibitors collected from literature used for generating both Pharmacophore and QSAR models, along with their fitness scores and 3D QSAR predicted activity.

**Supplementary Table S2.** Enrichment report of the 20 generated pharmacophore hypotheses.

**Supplementary Figure 1. (a)** The alignment of the most active compound **4** and **(b)** least active compound **11** to the **ADDRR_3** hypothesis with its pharmacophoric features, one H-bond acceptor (A5), one H-bond donor (D6), and three aromatic rings (R13, R14, R15), displayed individually with pink, cyan and yellow colour cubic sites.

**Supplementary Figure 2.** 2D Ligand interactions diagram of the potential hits **6**, **7**, **8**, **9**, and **10** with DprE1 (PDB:4KW5).

**Supplementary Figure 3.** 2D Ligand interactions diagram of the DprE1 clinical candidates with DprE1 (PDB:4KW5).

**Supplementary Figure 4.** DprE1 protein-ligand RMSD plot of **(a)** hit **5** **(c)** hit **9,** and RMSF plot of DprE1 in the presence of **(b)** hit **5** **(d)** hit **9**.

**Supplementary Figure 5.** Protein-ligand contacts histogram of **(a)** hit 5, **(c)** hit **9,** and Ligand-protein interactions of **(b)** hit **5,** **(d)** hit **9**.

**Supplementary Figure 6.** Superimposed pose of the co-crystallized inhibitor of DprE1 protein (PDB ID: 4KW5) for the validation of docking protocol.

**Supplementary Table S1.** Structures and their reported biological activities (IC_50_) of 39 azaindole scaffold based DprE1 inhibitors collected from literature used for generating both Pharmacophore and QSAR models, along with their fitness scores and 3D QSAR predicted activity.

| **SL No.** | **Structure of the collected DprE1 inhibitors** | **Reported Biological activity (IC_50_)** | **Calculated Biological activity (p^IC50^)** | **Pharm**  **set** | **Fitness score** | **QSAR set** | **QSAR predicted activity** |
| --- | --- | --- | --- | --- | --- | --- | --- |
| 1 |  | 0.014 | 7.853 | Moderately active | 2.390 | Test | 7.5705 |
| 2 |  | 0.010 | 8.000 | Moderately active | 2.223 | Training | 7.93217 |
| 3 |  | 0.005 | 8.301 | Active | 2.737 | Training | 8.30476 |
| 4 |  | 0.003 | 8.522 | Active | 3.000 | Training | 8.06618 |
| 5 |  | 0.010 | 8.000 | Moderately active | 2.354 | Test | 7.75191 |
| 6 |  | 0.010 | 8.000 | Moderately active | 2.132 | Training | 7.93331 |
| 7 |  | 0.006 | 8.221 | Active | 2.875 | Test | 8.04861 |
| 8 |  | 0.007 | 8.154 | Active | 2.845 | Training | 8.13668 |
| 9 |  | 1 | 6.000 | Inactive | 2.832 | Test | 6.60928 |
| 10 |  | 5.5 | 5.259 | Inactive | 2.292 | Training | 5.35652 |
| 11 |  | 7.9 | 5.102 | Inactive | 2.662 | Training | 4.499921 |
| 12 |  | 0.021 | 7.677 | Moderately active | 2.602 | Training | 7.79853 |
| 13 |  | 0.083 | 7.080 | Moderately active | 2.203 | Training | 6.75496 |
| 14 |  | 2.5 | 5.602 | Inactive | 1.971 | Training | 5.76049 |
| 15 |  | 0.014 | 7.853 | Moderately active | 2.353 | Training | 7.86795 |
| 16 |  | 0.037 | 7.431 | Moderately active | 2.203 | Training | 7.65423 |
| 17 |  | 0.020 | 7.698 | Moderately active | 2.007 | Training | 7.5395 |
| 18 |  | 0.090 | 7.045 | Moderately active | 2.111 | Training | 6.91259 |
| 19 |  | 0.004 | 8.397 | Active | 2.851 | Test | 8.63571 |
| 20 |  | 0.004 | 8.397 | Active | 2.784 | Training | 8.28714 |
| 21 |  | 0.007 | 8.154 | Active | 2.584 | Training | 8.06636 |
| 22 |  | 0.019 | 7.721 | Moderately active | 2.429 | Test | 7.66572 |
| 23 |  | 0.028 | 7.552 | Moderately active | 2.197 | Test | 7.66669 |
| 24 |  | 0.021 | 7.677 | Moderately active | 2.148 | Training | 7.76107 |
| 25 |  | 0.013 | 7.886 | Moderately active | 2.110 | Training | 8.12931 |
| 26 |  | 0.009 | 8.045 | Active | 2.957 | Test | 8.06291 |
| 27 |  | 0.019 | 7.721 | Moderately active | 2.180 | Training | 7.68703 |
| 28 |  | 0.007 | 8.154 | Active | 2.923 | Training | 8.06269 |
| 29 |  | 0.003 | 8.522 | Active | 1.919 | Test | 8.40559 |
| 30 |  | 0.043 | 7.366 | Moderately active | 2.073 | Test | 7.90247 |
| 31 |  | 0.005 | 8.301 | Active | 2.785 | Training | 8.28465 |
| 32 |  | 0.005 | 8.301 | Active | 2.880 | Training | 8.43803 |
| 33 |  | 0.017 | 7.769 | Moderately active | 2.150 | Training | 8.02563 |
| 34 |  | 0.011 | 7.958 | Moderately active | 2.228 | Training | 8.06385 |
| 35 |  | 0.015 | 7.823 | Moderately active | 2.210 | Training | 7.84109 |
| 36 |  | 0.022 | 7.657 | Moderately active | 2.192 | Training | 7.81838 |
| 37 |  | 0.2 | 6.698 | Inactive | 2.923 | Training | 7.00809 |
| 38 |  | 0.013 | 7.886 | Moderately active | 2.094 | Training | 7.63115 |
| 39 |  | 0.012 | 7.920 | Moderately active | 2.063 | Test | 8.0944 |
| 40 |  | 0.019 | 7.721 | Moderately active | 1.929 | Test | 7.16635 |

**Supplementary Table S2.** Enrichment report of the 20 generated pharmacophore hypotheses.

| Serial  No. | Hypothesis | Phase Hypo Score | EF% | BEDROC 160.9 | ROC | AUAC | Avg outranking decoys | Matches |
| --- | --- | --- | --- | --- | --- | --- | --- | --- |
| 1 | AAADR_1 | 1.29 | 91.91 | 1.00 | 1.00 | 0.99 | 0.00 | 5 of 5 |
| 2 | ADRRR_1 | 1.33 | 91.91 | 0.99 | 1.00 | 0.99 | 0.09 | 5 of 5 |
| 3 | ADRRR_2 | 1.32 | 82.72 | 0.98 | 1.00 | 0.99 | 0.36 | 5 of 5 |
| 4 | ADRRR_3 | 1.33 | 91.91 | 1.00 | 1.00 | 0.99 | 0.00 | 5 of 5 |
| 5 | ADRRR_4 | 1.19 | 73.53 | 0.88 | 1.00 | 0.99 | 1.64 | 5 of 5 |
| 6 | AADRR_1 | 1.32 | 91.91 | 0.99 | 1.00 | 0.99 | 0.09 | 5 of 5 |
| 7 | AADRR_2 | 1.31 | 82.72 | 0.98 | 1.00 | 0.99 | 0.27 | 5 of 5 |
| 8 | AADRR_3 | 1.31 | 91.91 | 1.00 | 1.00 | 0.99 | 0.00 | 5 of 5 |
| 9 | AADRR_4 | 1.30 | 91.91 | 1.00 | 1.00 | 0.99 | 0.00 | 5 of 5 |
| 10 | AADRR_5 | 1.28 | 91.91 | 0.97 | 1.00 | 0.99 | 1.00 | 5 of 5 |
| 11 | ADRR_1 | 1.30 | 82.72 | 0.98 | 1.00 | 0.99 | 0.27 | 4 of 4 |
| 12 | ADRR_2 | 1.30 | 91.91 | 0.99 | 1.00 | 0.99 | 0.09 | 4 of 4 |
| 13 | ADRR_3 | 1.30 | 82.72 | 0.98 | 1.00 | 0.99 | 0.36 | 4 of 4 |
| 14 | ADRR_4 | 1.30 | 91.91 | 0.99 | 1.00 | 0.99 | 0.18 | 4 of 4 |
| 15 | ADRR_5 | 1.29 | 82.72 | 0.96 | 1.00 | 0.99 | 0.73 | 4 of 4 |
| 16 | ADRR_6 | 1.30 | 91.91 | 1.00 | 1.00 | 0.99 | 0.00 | 4 of 4 |
| 17 | ARRR_1 | 1.30 | 82.72 | 0.98 | 1.00 | 0.99 | 0.27 | 4 of 4 |
| 18 | ARRR_2 | 1.29 | 73.53 | 0.95 | 1.00 | 0.99 | 0.82 | 4 of 4 |
| 19 | DRRR_1 | 1.31 | 82.72 | 0.98 | 1.00 | 0.99 | 0.27 | 4 of 4 |
| 20 | DRRR_2 | 1.31 | 91.91 | 1.00 | 1.00 | 0.99 | 0.00 | 4 of 4 |


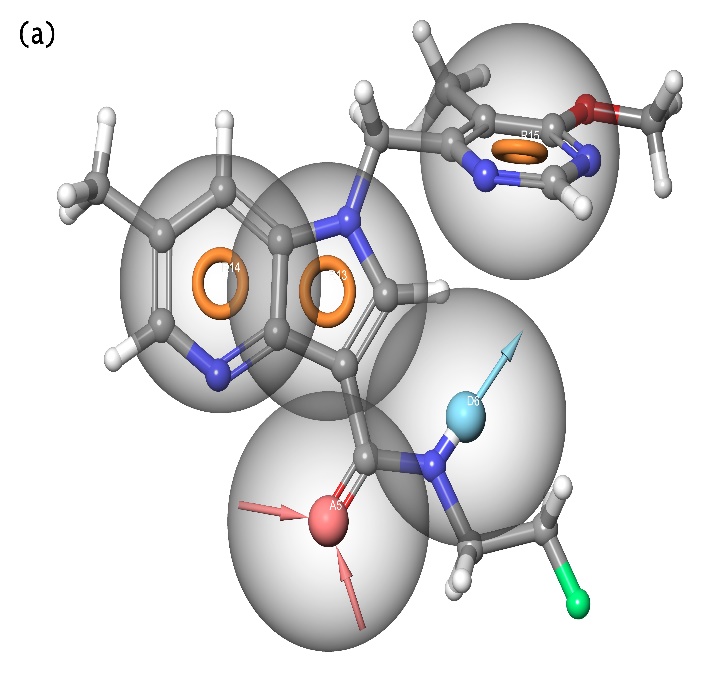

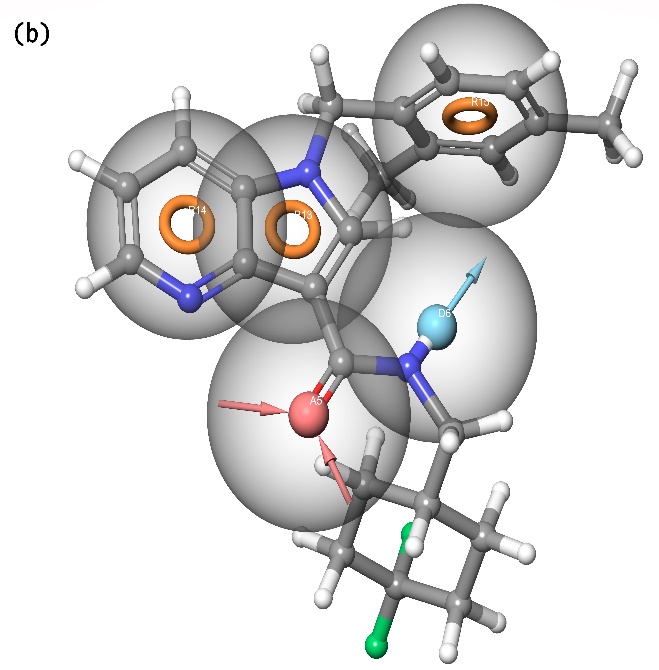


**Supplementary Figure 1. (a)** The alignment of the most active compound **4** and **(b)** least active compound **11** to the **ADDRR_3** hypothesis with its pharmacophoric features, one H-bond acceptor (A5), one H-bond donor (D6), and three aromatic rings (R13, R14, R15), displayed individually with pink, cyan and yellow colour cubic sites.


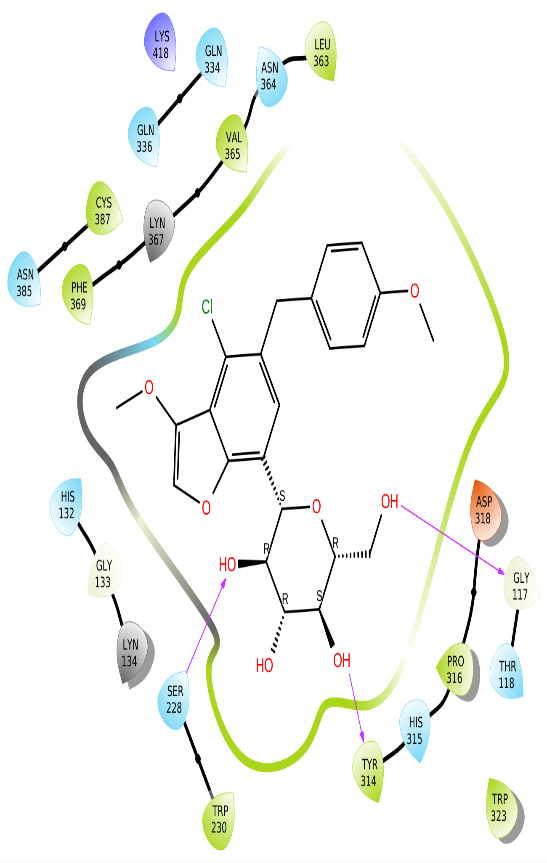

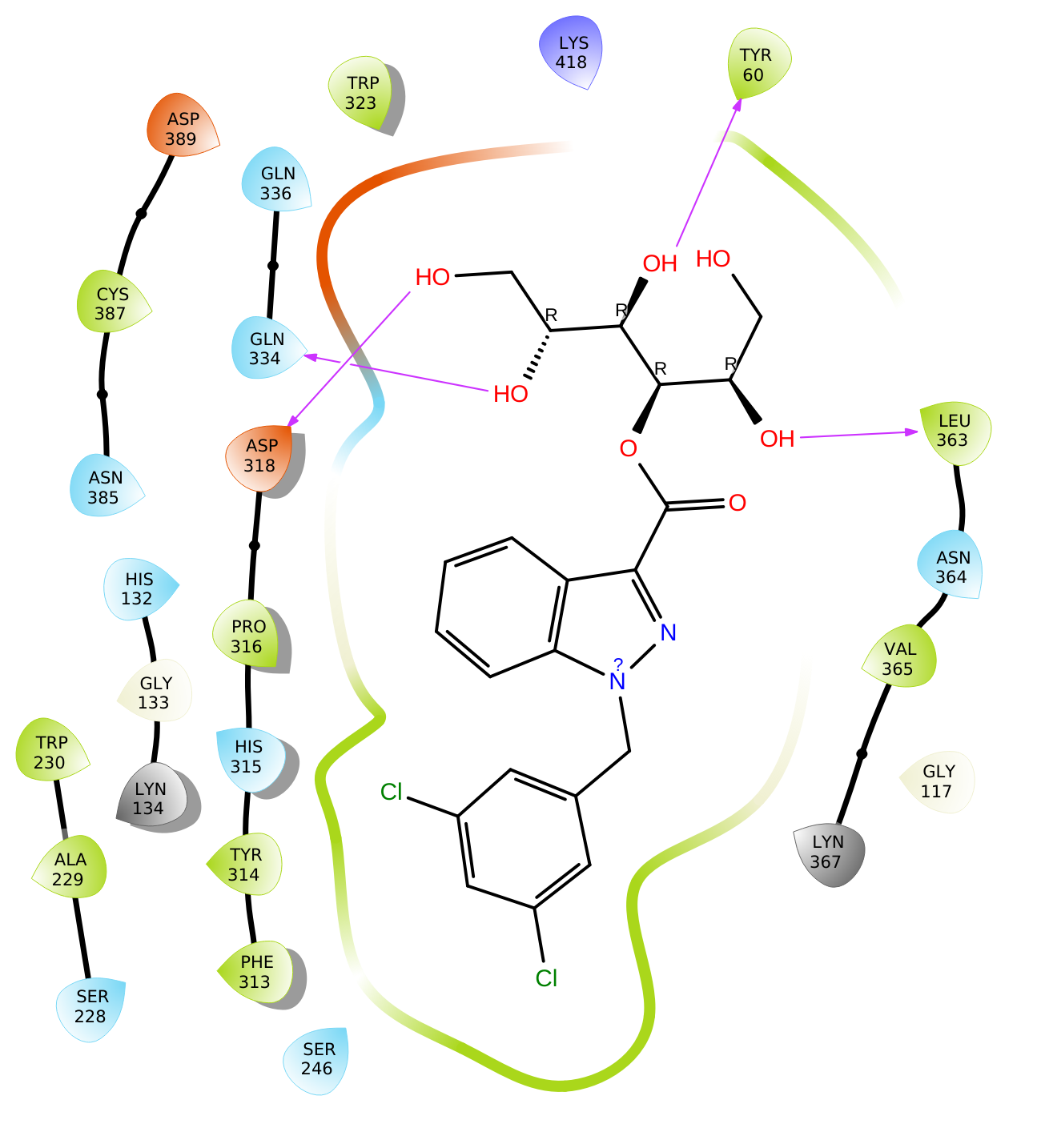

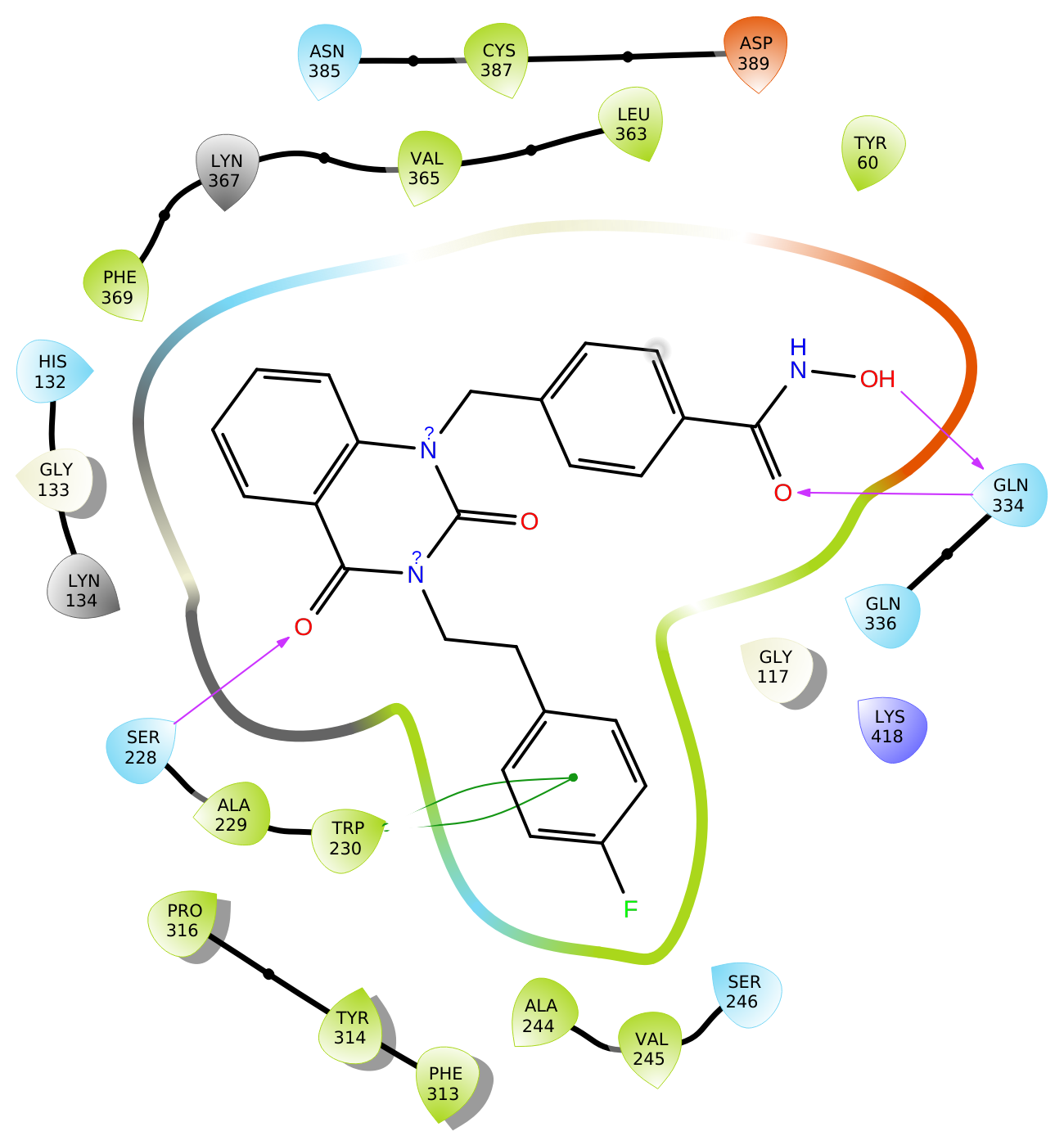


**Hit 8**

**Hit 7**

**Hit 6**


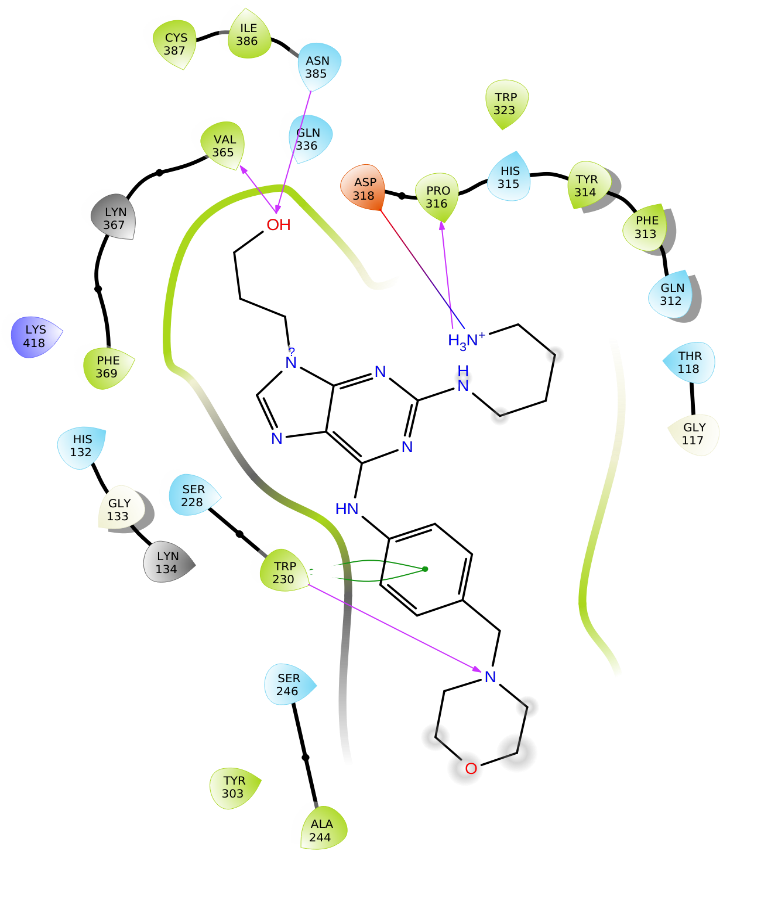

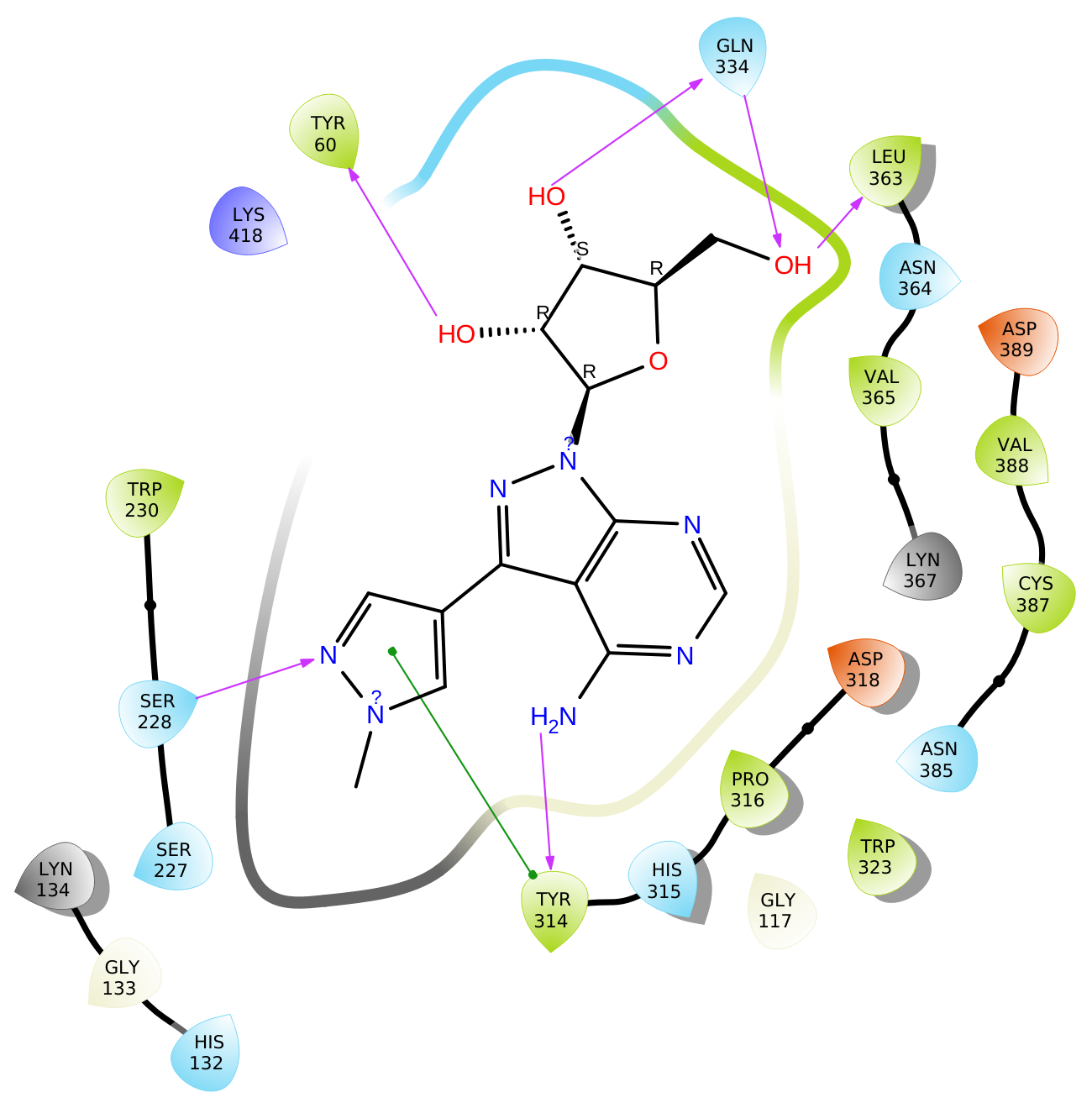


**Hit 9**

**Hit 10**

**Supplementary Figure 2.** 2D Ligand interactions diagram of the potential hits **6**, **7**, **8**, **9**, and **10** with DprE1 (PDB:4KW5).


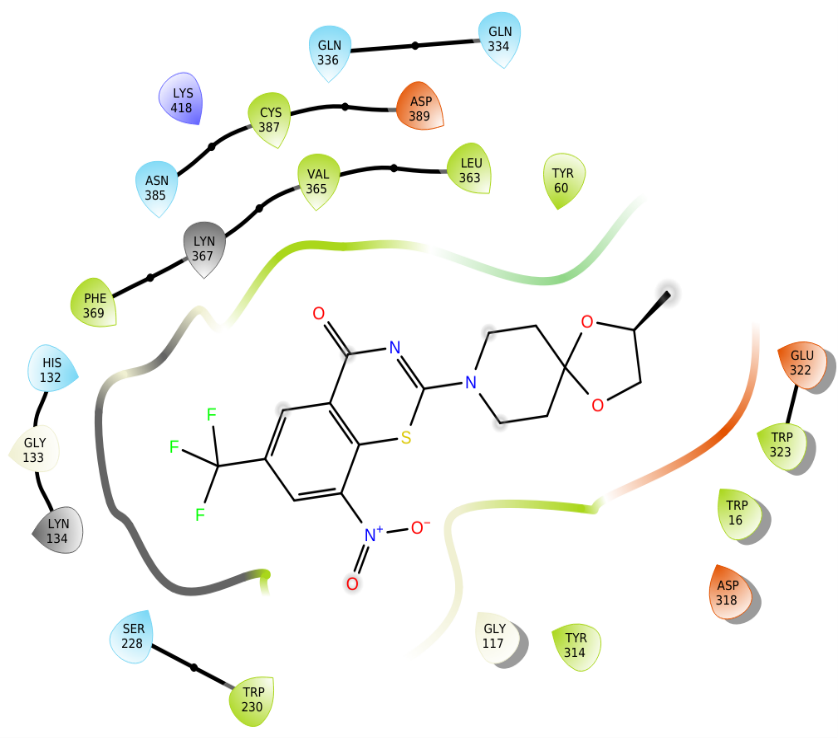


**BTZ-043**


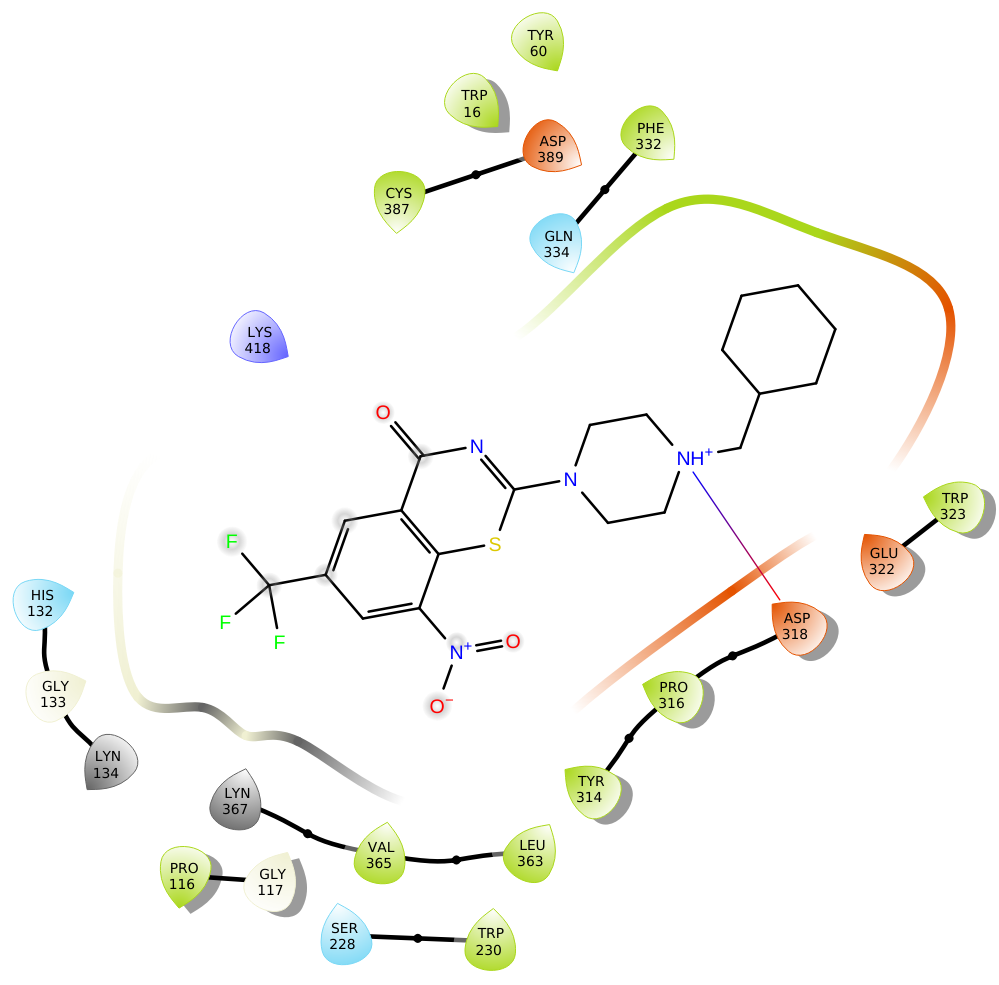


**PBTZ-169**


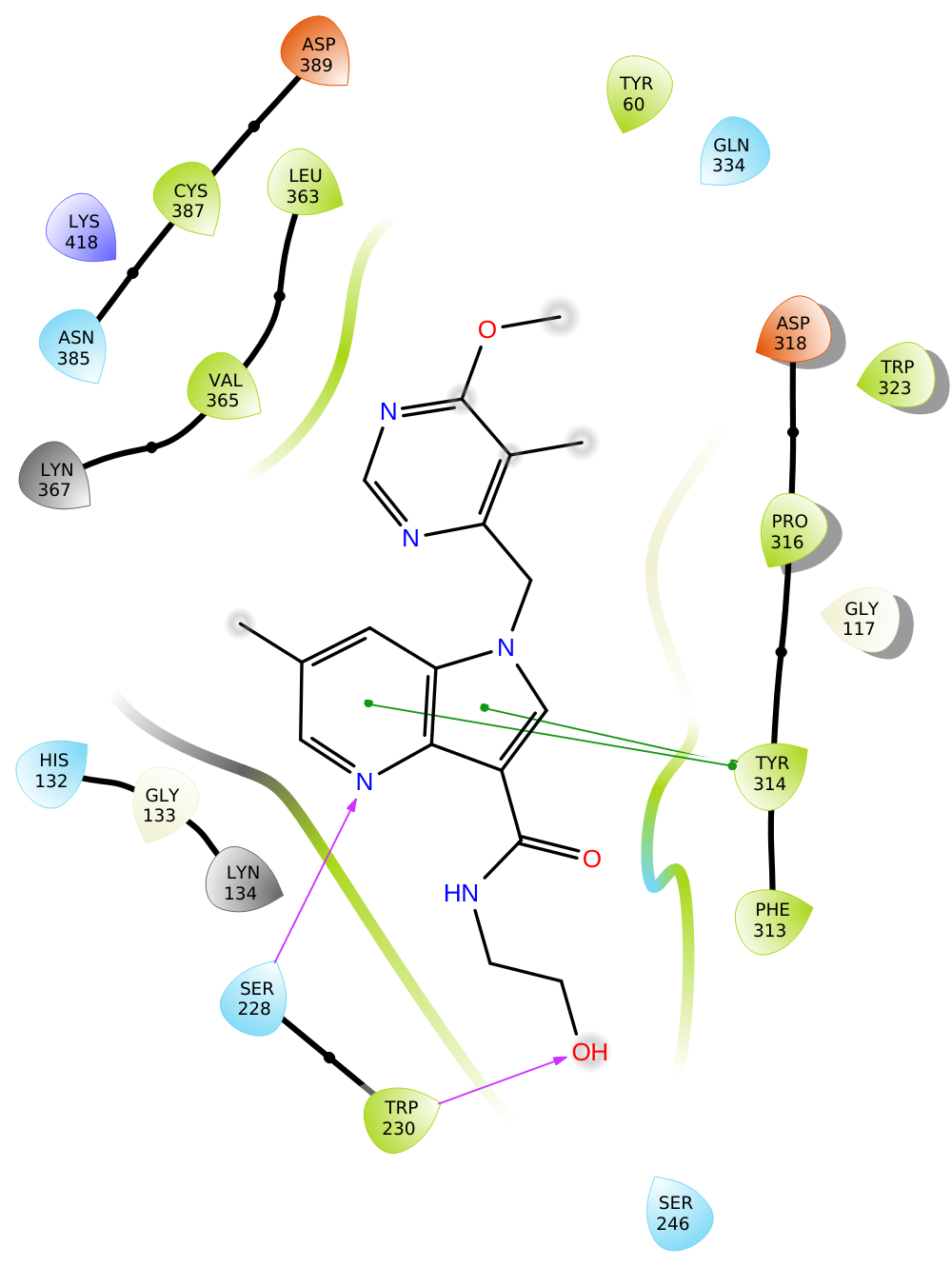


**TBA-7371**


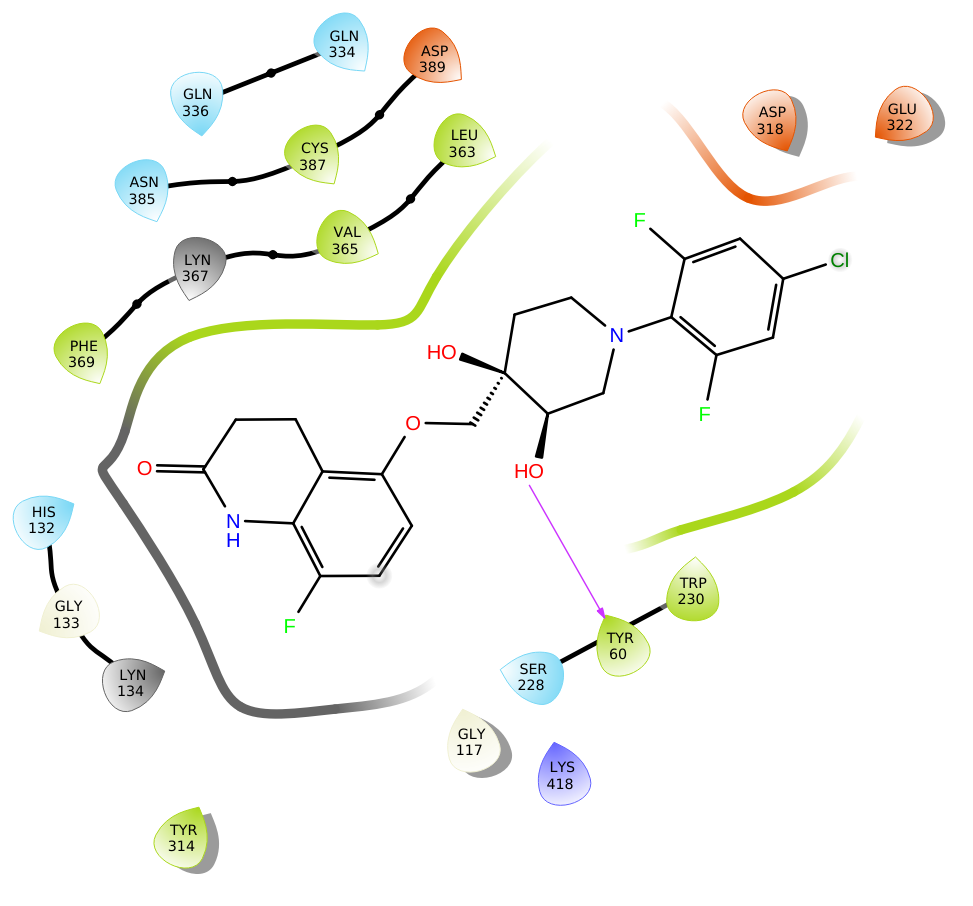


**OPC-167832**

**Supplementary Figure 3.** 2D Ligand interactions diagram of the DprE1 clinical candidates with DprE1 (PDB:4KW5).


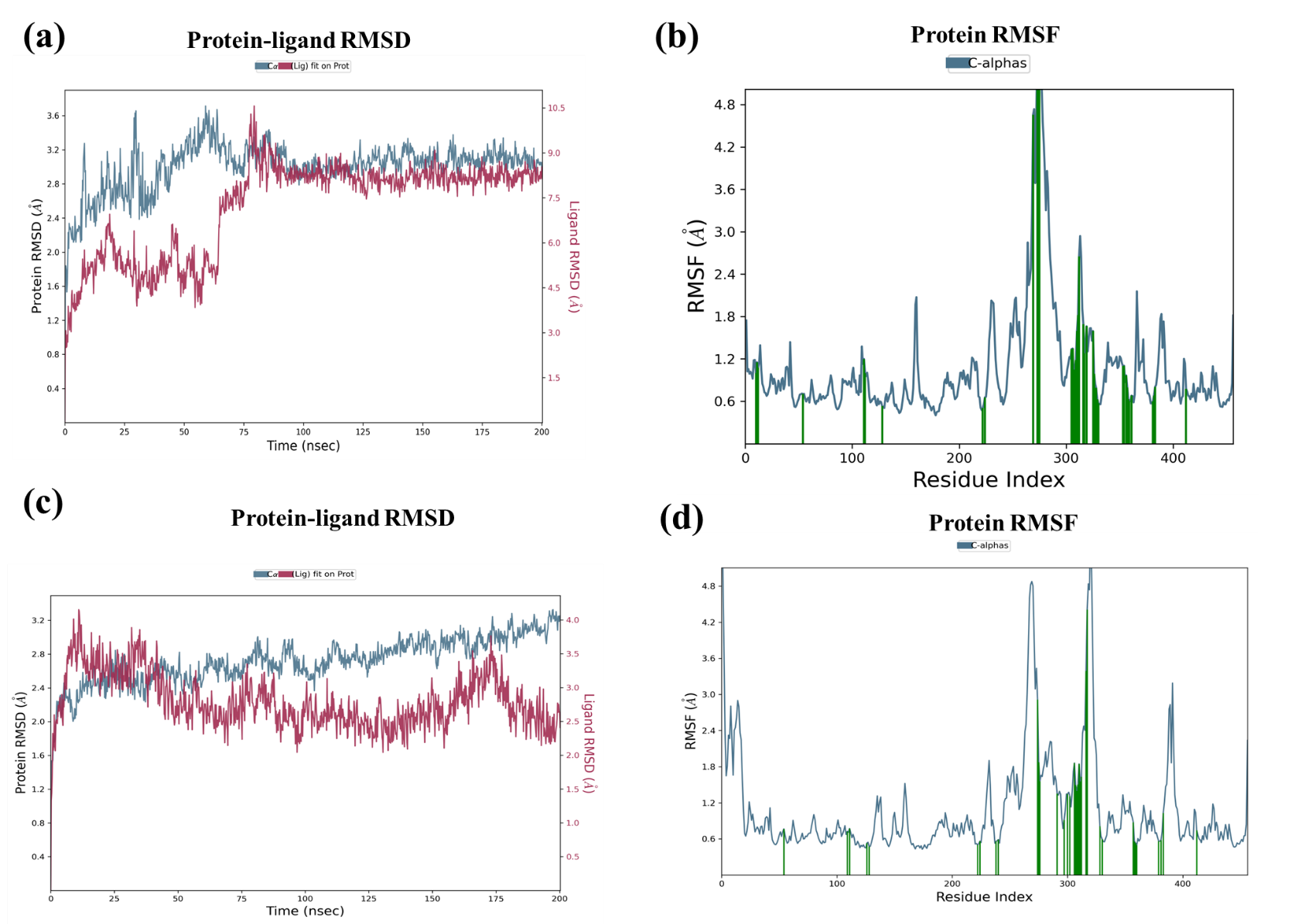


**Supplementary Figure 4.** DprE1 protein-ligand RMSD plot of **(a)** hit **5** **(c)** hit **9**, and RMSF plot of DprE1 in the presence of **(b)** hit **5** **(d)** hit **9**.


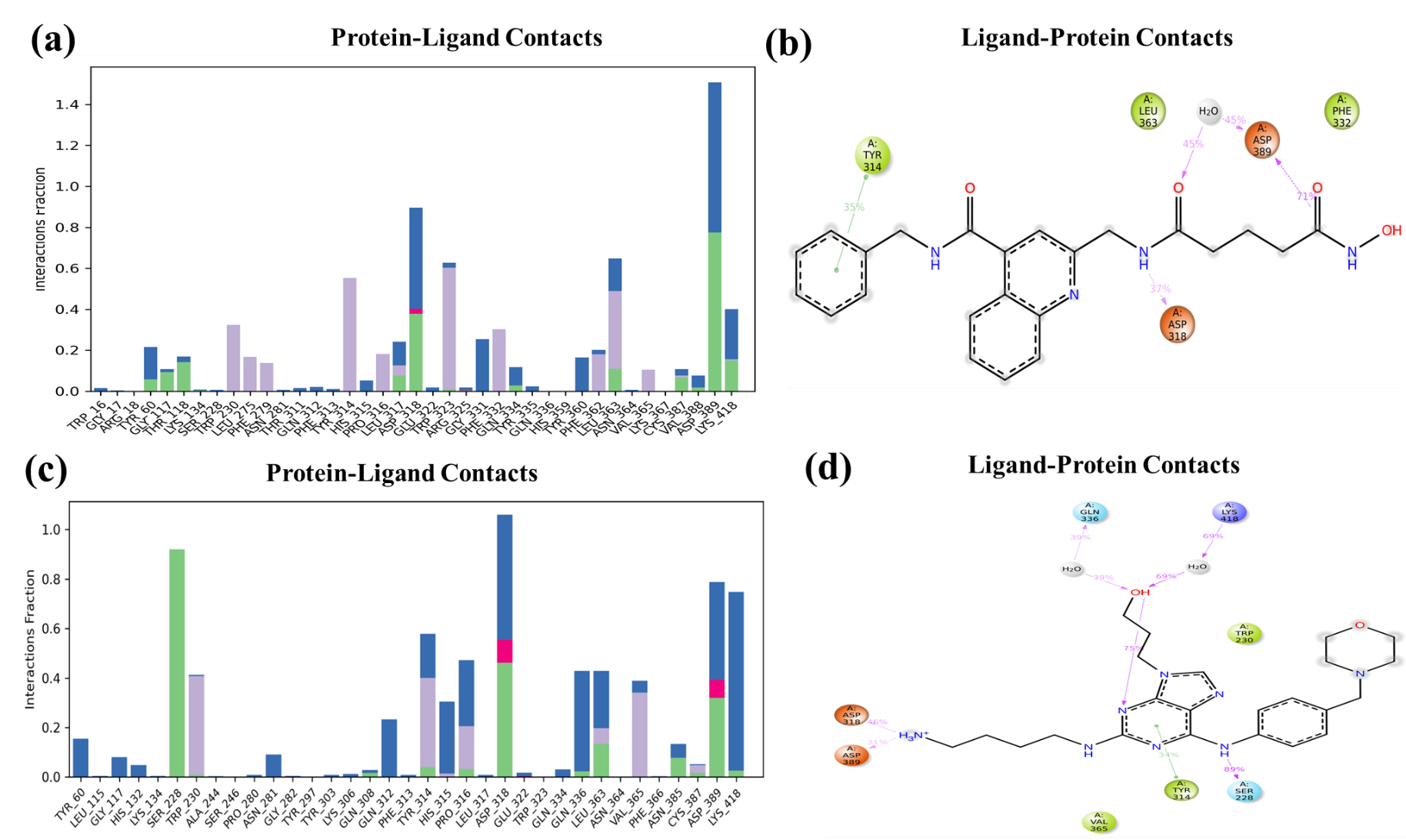


**Supplementary Figure 5.** Protein-ligand contacts histogram of **(a)** hit 5, **(c)** hit **9**, and Ligand-protein interactions of **(b)** hit **5**, **(d)** hit **9**.


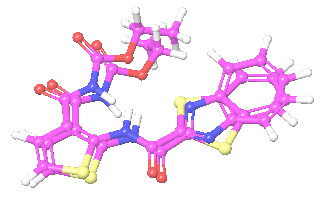


**Supplementary Figure 6.** Superimposed pose of the co-crystallized inhibitor of DprE1 protein (PDB ID: 4KW5) for the validation of docking protocol.
